# Albuminuria is associated with increased risk of dementia, independent of eGFR: The SCREAM project

**DOI:** 10.1111/joim.70022

**Published:** 2025-09-23

**Authors:** Li Luo, Ron T. Gansevoort, Lyanne M. Kieneker, Yuanhang Yang, Alessandro Bosi, Rudolf A. de Boer, Casper F. M. Franssen, Maria Eriksdotter, Juan‐Jesus Carrero, Hong Xu

**Affiliations:** ^1^ Department of Nephrology University Medical Center Groningen University of Groningen Groningen The Netherlands; ^2^ Department of Medical Epidemiology and Biostatistics Karolinska Institutet Stockholm Sweden; ^3^ Institute of Environmental Medicine Karolinska Institutet Stockholm Sweden; ^4^ Erasmus MC Thorax Center Department of Cardiology Cardiovascular Institute Rotterdam The Netherlands; ^5^ Division of Clinical Geriatrics Department of Neurobiology Care Sciences and Society Karolinska Institutet Stockholm Sweden; ^6^ Theme Inflammation and Aging Karolinska University Hospital Stockholm Sweden; ^7^ Division of Nephrology Department of Clinical Sciences Karolinska Institutet Danderyd Hospital Stockholm Sweden

**Keywords:** albuminuria, Alzheimer's dementia, chronic kidney disease, mixed dementia, vascular dementia

## Abstract

**Background:**

The association between albuminuria and dementia has been insufficiently studied, possibly due to not considering dementia subtypes, the interplay with estimated glomerular filtration rate (eGFR), and the use of varying albuminuria measurement techniques.

**Objectives:**

This study aimed to investigate the eGFR‐independent risk of all‐cause and type‐specific dementia associated with albuminuria, measured by the urine albumin‐creatinine ratio (ACR) and dipstick.

**Methods:**

The main analysis included 132,869 subjects aged ≥65 years without a history of dementia and with at least one ACR test from the Stockholm Creatinine Measurements (SCREAM) project between 2006 and 2019. The primary and secondary outcomes were the incidence of all‐cause dementia and type‐specific dementia, respectively. Cox regression models were used to calculate hazard ratios (HRs, 95% CIs).

**Results:**

During a median follow‐up of 3.9 (interquartile ranges, 1.8–7.1) years, 9435 (7%) subjects developed incident dementia. After multivariable adjustments, including eGFR, an ACR level of 30–299 and ≥300 mg/g was associated with a 25% (HR, 1.25; 95% CI, 1.19–1.31) and a 37% (HR, 1.37; 95% CI, 1.23–1.51) higher risk of developing all‐cause dementia, respectively, compared to an ACR level of <30 mg/g. Higher ACR levels were also associated with an increased risk of mixed, vascular, and unspecified dementia, but not with Alzheimer's disease. These findings were robust in subjects with at least one dipstick proteinuria test.

**Conclusion:**

Increased albuminuria is associated with a higher risk of all‐cause dementia, particularly mixed, vascular, and unspecified dementia, independent of baseline eGFR and generalizable across different clinical pathways of albuminuria testing.

## Graphical abstract

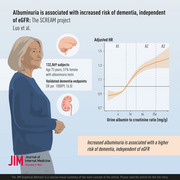

## Introduction

Chronic kidney disease (CKD) is associated with dysfunction of multiple organs. Recent studies have suggested that CKD could also be a risk factor for dementia [1]. Although estimated glomerular filtration rate (eGFR) and albuminuria are two key markers to define and stage CKD, previous studies on the association between CKD and dementia focused more on eGFR than on albuminuria [2]. Of note, dementia is not yet curable, and rising efforts are dedicated to identifying risk factors in the prevention setting. Because albuminuria is modifiable by several established treatment options, the potential of albuminuria as a risk factor for dementia is therefore clinically relevant, which in succession may benefit the prevention of dementia [3].

Among those few studies focusing on the association between albuminuria and dementia, the risk of different dementia subtypes associated with albuminuria is insufficiently studied, probably due to the limited sample size [4, 5, 6]. Separate investigation into different dementia subtypes is crucial because dementia is a heterogeneous term covering subtypes that can have distinct risk factors, disease management, and progression patterns [7, 8, 9]. Another critical consideration is that there is a paucity of evidence on how albuminuria interacts with eGFR in the estimation of dementia risk [10]. Furthermore, it is largely unknown whether the association between albuminuria and dementia is altered in the context of different albuminuria testing pathways, such as the most common techniques of fully quantitative urine albumin‐creatinine ratio (ACR) and semiquantitative dipstick proteinuria.

This study, therefore, aimed to investigate the eGFR‐independent risk of all‐cause and type‐specific dementia associated with albuminuria. To address the gaps above in the current evidence, we examined the risk of type‐specific dementia associated with albuminuria, evaluated the effects of the interplay of albuminuria and eGFR in the albuminuria dementia association, and quantified albuminuria by ACR and dipstick proteinuria in a large Swedish population.

## Materials and methods

### Study population

We analyzed data from the Stockholm Creatinine Measurements (SCREAM) project, a cohort comprising data on all residents accessing healthcare in Stockholm, Sweden, during 2006–2019 [11]. Laboratory data were linked with regional and national administrative registries for records on healthcare utilization, prescribed medications, socioeconomic factors, validated kidney replacement therapy endpoints, and follow‐up for death, with essentially no loss to follow‐up. For our main analyses, we selected subjects with no prior history of all‐cause dementia who had undergone at least one ACR test. For sensitivity analyses, we included similar subjects who had at least one dipstick proteinuria test. Subjects were selected on the basis of the date of the first‐encountered ACR or dipstick test per patient (if more than one was available). As a next step, we assessed the presence of a creatinine‐based eGFR value within 6 months before or after the selected albuminuria test, defining this as concomitant eGFR. The baseline (index) date of the cohorts was set as the latest of the albuminuria or eGFR test dates. Exclusion criteria, covariate assessment, and follow‐up initiation were all conducted at the index date. Subjects aged <65 years were excluded, given that 96% of dementia diagnoses occur in those aged ≥65 years [12], and that SCREAM captured >90% of all subjects aged ≥65 years in the region [11]. Other exclusion criteria included a history of all‐cause dementia, maintaining kidney replacement therapy, death on the same day as the baseline assessment, and having missing data on concurrent eGFR or the highest attained education (Fig. S1). The study was approved by the regional ethics committee in Stockholm, Sweden.

### Exposure and covariates

All laboratory measurements were conducted using routine methods at the clinical laboratories of the Stockholm region. ACR was calculated by dividing urinary albumin by urinary creatinine, expressed in mg/mmol (to convert to mg/g, multiply by 8.84). ACR values were further classified into KDIGO categories A1–A3 [13].

Baseline covariates consisted of age, sex, highest attained education, lifestyle factors (tobacco abuse, alcohol abuse, and diagnosed obesity), comorbidities (diabetes, hypertension, congestive heart failure, myocardial infarction, atrial fibrillation, stroke, cancer, depression, and hearing loss), ongoing medications [the use of angiotensin‐converting enzyme inhibitors/angiotensin receptor blockers (ACEi/ARBs), beta blockers, calcium channel blockers, nonsteroidal anti‐inflammatory drugs, and statins], and eGFR. The highest attained education was grouped into three levels, as obtained from the longitudinal integrated database for health insurance and labor market studies (LISA) register [14]. Ongoing medications were ascertained by Anatomical Therapeutic Chemical codes. Cancer data were obtained by linkage to the Swedish Cancer Registry [15]. Lifestyle factors and other comorbidities were determined with ICD‐10 codes. The definitions of hypertension, diabetes, and depression were further supplemented by data on the use of corresponding medications (Table S1). eGFR was derived from serum/plasma creatinine using the 2009 CKD Epidemiology Collaboration (CKD‐EPI) creatinine equation [16]. The 2021 CKD‐EPI equation is not currently adopted in Sweden or across Europe [17, 18]. To better reflect kidney function, we restricted to use creatinine measurements obtained in the outpatient setting, and all creatinine tests were standardized to isotope dilution mass spectrometry standards. Ethnicity data are not allowed to be documented in Sweden by law, and therefore, all subjects were assumed to be white.

### Follow‐up and study outcomes

The primary outcome was a clinical diagnosis of dementia, ascertained primarily from registration in the Swedish Dementia Register (SveDem) [19], followed by the presence of ICD‐10‐based diagnoses of dementia in any source of Swedish healthcare and the dispensation of specific anti‐dementia drugs (donepezil, rivastigmine, galantamine, and/or memantine) in any Swedish pharmacy. The secondary outcomes included specific dementia types as recorded in SveDem: (1) Alzheimer's disease (AD), (2) mixed AD, (3) vascular dementia (VaD), (4) dementia with Lewy body (DLB) and Parkinson's disease with dementia (PDD), (5) frontotemporal dementia (FTD), (6) unspecified dementia, and (7) other types such as cortico‐basal syndrome or alcohol‐related dementia [20]. The etiology of dementia was primarily obtained from registration in SveDem. ICD‐10 coding was also used to classify subtypes only when there were no available dementia records in SveDem. Details of outcome ascertainment are presented in Table S2. Subjects were censored at the end of follow‐up (December 31, 2018), death, or emigration from the region, whichever occurred first. Death data were retrieved from the National Board of Health and Welfare's Cause‐of‐Death Register (https://www.socialstyrelsen.se).

### Statistical analyses

Continuous variables are presented as medians with interquartile ranges (IQR) regardless of normal or skewed distribution. Categorical variables are summarized by counts and proportions of their categories.

Incidence rates (IRs) per 1000 person‐years with 95% confidence intervals (95% CIs) were calculated using the Clopper–Pearson exact method [21]. The 10‐year cumulative incidence was estimated using the Aalen–Johansen estimator, accounting for mortality as a competing event [22]. Cox proportional hazards regression models were used to estimate the association between baseline KDIGO albuminuria categories and the incidence of all‐cause dementia and dementia subtypes. Results are reported as hazard ratios (HRs) with 95% CIs. Model 1 was adjusted for age. In model 2, we calculated HRs adjusted for sex, education, tobacco abuse, alcohol abuse, diagnosed obesity, cancer, depression, and hearing loss. In model 3, we additionally adjusted for confounders relevant to vascular dysfunction and baseline eGFR. With this modeling strategy, we want to specifically check possible confounding effects of vascular dysfunction in the association between albuminuria and dementia. We found no evidence for multicollinearity, considering that generalized variance‐inflation factors (GVIFs) of variables included in the fully adjusted model were all <10 (Table S3) [23]. We also found no indication for major violations of the proportional hazards assumption, justified by no noticeable pattern in Schoenfeld residuals with time (Fig. S2). Additionally, restricted cubic splines were fitted to explore nonlinearity in the association of all‐cause dementia incidence with albuminuria as a continuous variable. HRs (95% CIs) for all‐cause dementia incidence according to KDIGO combined albuminuria and eGFR global risk strata (<30, 30–59, 60–89, and >90 m/min/1.73 m^2^) were also calculated using Cox regression models [13].

We conducted several sensitivity and supporting analyses. First, to examine the generalizability of the association between albuminuria and dementia across different albuminuria testing pathways, we replicated the main analyses in subjects who had at least one dipstick proteinuria test. Dipstick proteinuria was assessed by an automated urine analyzer and recorded as negative, 1+, 2+, and 3+ [24]. Second, to explore the possibility of reverse causation and detection bias, we did a 1‐year landmark analysis by excluding subjects with a follow‐up time of ≤1 year, deducting 1 year from the follow‐up time of the remaining included subjects, and remodeling the associations as such to determine a new baseline at 1 year to initiate study follow‐up. Third, to possible unmeasured confounding, we repeated our analysis using cataracts as a negative control outcome (i.e., cataracts are not plausibly affected by albuminuria but share the same set of confounders or sources of bias in the association of albuminuria with dementia) [25]. Fourth, to assess whether using the 2021 CKD‐EPI and the revised Lund–Malmo equations to calculate eGFR influences the estimates of the albuminuria dementia association, we applied these equations to calculate eGFR and reanalyzed the associations [26, 27]. Fifth, to verify stability and robustness on estimating the associations between albuminuria and all‐cause dementia, we redefined all‐cause dementia to include only AD, mixed AD, VaD, and unspecified dementia and remodeled the associations. Sixth, as the incidence of dementia subtypes varies with age, some subtypes, for example, early onset dementia and FTD [28], could occur before the age <65; thus, we included subjects aged ≥50 years and estimated the associations. Seventh, to evaluate the potential influence of increasing national coverage of dementia case ascertainment, we repeated the analyses starting the study follow‐up from 2012, when SveDem achieved to capture 36% incident dementia cases [19].

Furthermore, to detect possible effect modification by baseline age (<75 and ≥75 years old), sex (female/male), hypertension (presence/absence), diabetes (presence/absence), and eGFR (<60 and ≥60 mL/min/1.73 m^2^), we fitted Cox models containing both main effects and the cross‐product terms with albuminuria. Additionally, to investigate the influence of mortality as a competing event on the cumulative incidence of all‐cause dementia, we performed competing risk analysis using Fine and Gray subdistribution hazard regression [29].

*p* values are two‐tailed. A *p* value of <0.05 is considered statistically significant. Analyses were conducted with R (version 4.2.3, R Foundation for Statistical Computing, Vienna, Austria). R packages of survival::coxph() and cmprsk::crr() were used to perform Cox regression and competing risk analyses, respectively, with default settings applied except for cohort‐specific parameters.

## Results

### Baseline characteristics

After applying the inclusion and exclusion criteria, 138,688 subjects with data on at least 1 ACR test, age ≥65 years, and no dementia history were included (Table 1). Their mean age was 73.0 (69.0, 79.0) years, and 50.5% were female. Median baseline ACR was 9.7 (IQR, 4.6, 29.2) mg/g, and mean baseline eGFR was 74.7 (59.9, 86.3) mL/min/1.73 m^2^. Among subjects with ACR >30 mg/g, 89.7% had hypertension, 41.6% had diabetes, and 61.9% used ACEi/ARBs.

**Table 1:** Baseline characteristics of eligible subjects overall and stratified by KDIGO albuminuria categories.

	Overall	KDIGO albuminuria categories (mg/g)		
		A1 <30	A2 30–299	A3 ≥300
No. of subjects	132,869	102,831	24,908	5130
**Demographics**				
Age, years	73.0 (69.0, 79.0)	73.0 (68.0, 78.0)	76.0 (70.0, 82.0)	76.0 (70.0, 82.0)
Age category, years				
<70	39,474 (29.7%)	32,793 (31.9%)	5526 (22.2%)	1155 (22.5%)
70–75	35,974 (27.1%)	29,216 (28.4%)	5613 (22.5%)	1145 (22.3%)
75–80	26,204 (19.7%)	20,032 (19.5%)	5142 (20.6%)	1030 (20.1%)
80–85	17,599 (13.2%)	12,419 (12.1%)	4272 (17.2%)	908 (17.7%)
≥85	13,618 (10.2%)	8371 (8.1%)	4355 (17.5%)	892 (17.4%)
Female	67,089 (50.5%)	54,435 (52.9%)	10,822 (43.4%)	1832 (35.7%)
Highest attained education				
Compulsory school	38,557 (29.0%)	28,019 (27.2%)	8637 (34.7%)	1901 (37.1%)
Secondary school	54,506 (41.0%)	42,448 (41.3%)	9949 (39.9%)	2109 (41.1%)
University	39,806 (30.0%)	32,364 (31.5%)	6322 (25.4%)	1120 (21.8%)
**Lifestyle**				
Tobacco abuse	2423 (1.8%)	1725 (1.7%)	555 (2.2%)	143 (2.8%)
Alcohol abuse	4811 (3.6%)	3466 (3.4%)	1074 (4.3%)	271 (5.3%)
Diagnosed obesity	10,343 (7.8%)	8037 (7.8%)	1918 (7.7%)	388 (7.6%)
**Kidney function**				
Albuminuria, median	9.7 (4.6, 29.2)	7.1 (4.1, 13.3)	59.2 (38.9, 106.1)	640.9 (403.1, 1294.2)
eGFR, mL/min/1.73 m^2^	74.7 (59.9, 86.3)	76.6 (63.2, 87.0)	67.8 (50.1, 83.5)	47.9 (27.6, 70.7)
eGFR category, mL/min/1.73 m^2^				
≥90	19,833 (14.9%)	16,452 (16.0%)	3031 (12.2%)	350 (6.8%)
60–89	79,477 (59.8%)	65,660 (63.9%)	12,367 (49.7%)	1450 (28.3%)
30–59	28,914 (21.8%)	19,419 (18.9%)	7598 (30.5%)	1897 (37.0%)
<30	4645 (3.5%)	1300 (1.3%)	1912 (7.7%)	1433 (27.9%)
**Comorbidities**				
Diabetes mellitus	43,858 (33.0%)	31,348 (30.5%)	10,175 (40.9%)	2335 (45.5%)
Hypertension	112,007 (84.3%)	85,054 (82.7%)	22,216 (89.2%)	4737 (92.3%)
Congestive heart failure	16,080 (12.1%)	9703 (9.4%)	5044 (20.3%)	1333 (26.0%)
Myocardial infarction	13,293 (10.0%)	9059 (8.8%)	3427 (13.8%)	807 (15.7%)
Atrial fibrillation	19,900 (15.0%)	12,997 (12.6%)	5700 (22.9%)	1203 (23.5%)
Stroke	11,779 (8.9%)	7978 (7.8%)	3044 (12.2%)	757 (14.8%)
Cancer	30,205 (22.7%)	22,159 (21.5%)	6609 (26.5%)	1437 (28.0%)
Depression	13,116 (9.9%)	10,379 (10.1%)	2317 (9.3%)	420 (8.2%)
Hearing loss	19,418 (14.6%)	14,991 (14.6%)	3738 (15.0%)	689 (13.4%)
**Medication**				
ACEi/ARBs	75,743 (57.0%)	57,152 (55.6%)	15,242 (61.2%)	3349 (65.3%)
Beta blockers	55,368 (41.7%)	40,081 (39.0%)	12,454 (50.0%)	2833 (55.2%)
Calcium channel blocker	41,080 (30.9%)	29,617 (28.8%)	9049 (36.3%)	2414 (47.1%)
NSAIDs	52,369 (39.4%)	39,189 (38.1%)	10,841 (43.5%)	2339 (45.6%)
Statins	52,810 (39.7%)	40,481 (39.4%)	10,114 (40.6%)	2215 (43.2%)

*Note*: Continuous variables are reported as median (interquartile range), and categorical variables are reported as *N* (%).

Abbreviations: ACEi, angiotensin‐converting enzyme inhibitor; ARB, angiotensin receptor blockers; eGFR, estimated glomerular filtration rate; KDIGO, Kidney Disease Improving Global Outcomes; NSAIDs, nonsteroidal anti‐inflammatory agent.

### Albuminuria and the risk of all‐cause dementia incidence

**Fig. 1:**
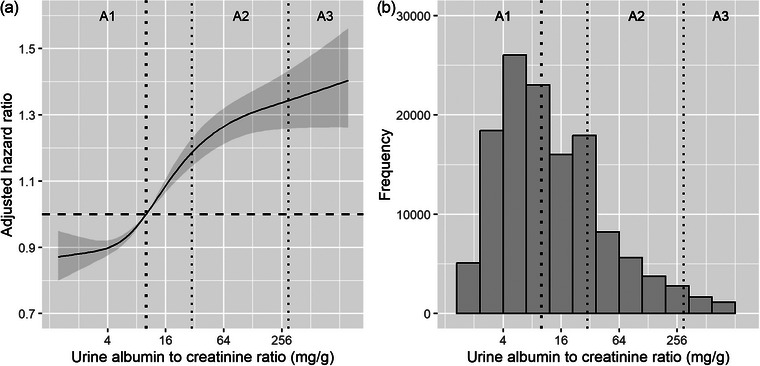
**Adjusted hazard ratio of albuminuria with the incidence of all‐cause dementia (panel a) and distribution of albuminuria (panel b)**. The spline shows the association of albuminuria with the incidence of all‐cause dementia. Data were fitted by Cox proportional hazards regression models based upon restricted cubic splines with 4 knots and adjusted for age, sex, education, tobacco abuse, alcohol abuse, diagnosed obesity, diabetes, hypertension, congestive heart failure, myocardial infarction, atrial fibrillation, stroke, cancer, depression, hearing loss, ACEi/ARBs, beta blockers, calcium channel blockers, NSAIDs, statins, and eGFR. The spline curves are truncated at the 1.0th and 99.0th percentiles of the distribution curve. The reference standard for albuminuria is 10 mg/g. The dashed lines represent 10, 30, and 300 mg/g. p value for the nonlinear association is p < 0.001. ACEi, angiotensin‐converting enzyme inhibitor; ARB, angiotensin receptor blockers; eGFR, estimated glomerular filtration rate; NSAIDs, nonsteroidal anti‐inflammatory agents.

During a median follow‐up of 3.9 (IQR, 1.8–7) years, 9435 (7%) subjects developed de novo all‐cause dementia. The 10‐year cumulative incidence of all‐cause dementia was 13.4% (95% CI, 13.1%–13.7%, Table 2). After a full adjustment including baseline eGFR, subjects with an ACR level of 30–299 mg/g and an ACR level of ≥300 mg/g had a 25% (HR, 1.25; 95% CI, 1.19–1.31) and 37% (HR, 1.37; 95% CI, 1.23–1.51) higher risk of developing all‐cause dementia, respectively, when compared to subjects with an ACR level of <30 mg/g. Additionally, when handled on a continuous scale, a doubling of ACR was associated with a higher risk of all‐cause dementia incidence in a nonlinear fashion (*p*_nonlinearity_ < 0.001, Fig. 1, Fig. S3).

**Table 2:** Associations of KDIGO albuminuria categories with the incidence of all‐cause dementia and type‐specific dementia.

KDIGO albuminuria categories (mg/g)	No. of events/participants	IR per 1000 PY (95% CI)	10‐year cumulative incidence (%, 95%CI)	Model 1, HR (95%CI)	*p* value	Model 2, HR (95%CI)	*p* value	Model 3, HR (95%CI)	*p* value
All‐cause dementia	9435/132,869	16.0 (15.6–16.3)	13.4 (13.1–13.7)						
A1 <30	6663/102,831	14.4 (14.0–14.7)	13.2 (12.8–13.5)	1.00 (ref.)		1.00 (ref.)		1.00 (ref.)	
A2 30–299	2341/24,908	21.7 (20.8–22.6)	14.7 (14.1–15.3)	1.25 (1.20–1.32)	<0.001	1.26 (1.20–1.32)	<0.001	1.25 (1.19–1.31)	<0.001
A3 ≥300	431/5130	21.6 (19.6–23.7)	11.0 (9.9–12.0)	1.35 (1.22–1.49)	<0.001	1.36 (1.23–1.50)	<0.001	1.37 (1.23–1.51)	<0.001
**Type‐specific dementia**									
Alzheimer's disease	1725/132,869	2.9 (2.8–3.1)	2.6 (2.4–2.7)						
A1 <30	1376/102,831	3.0 (2.8–3.1)	2.8 (2.6–2.9)	1.00 (ref.)		1.00 (ref.)		1.00 (ref.)	
A2 30–299	303/24,908	2.8 (2.5–3.1)	2.1 (1.8–2.4)	0.81 (0.72–0.92)	<0.001	0.87 (0.77–0.99)	<0.05	0.97 (0.85–1.10)	0.61
A3 ≥300	46/5130	2.3 (1.7–3.1)	1.3 (0.9–1.7)	0.71 (0.53–0.95)	<0.05	0.79 (0.59–1.07)	0.12	0.99 (0.74–1.34)	0.97
Mixed dementia	2293/132,869	3.9 (3.7–4.0)	3.5 (3.3–3.7)						
A1 <30	1659/102,831	3.6 (3.4–3.8)	3.5 (3.3–3.7)	1.00 (ref.)		1.00 (ref.)		1.00 (ref.)	
A2 30–299	542/24,908	5.0 (4.6–5.5)	3.7 (3.4–4.0)	1.18 (1.07–1.30)	<0.001	1.17 (1.06–1.29)	<0.001	1.16 (1.05–1.28)	<0.001
A3 ≥300	92/5130	4.6 (3.7–5.6)	2.6 (2.0–3.1)	1.17 (0.95–1.44)	0.15	1.16 (0.94–1.43)	0.18	1.19 (0.96–1.48)	0.12
Vascular dementia	2181/132,869	3.7 (3.5–3.8)	3.3 (3.2–3.5)						
A1 <30	1474/102,831	3.2 (3.0–3.3)	3.2 (3.0–3.3)	1.00 (ref.)		1.00 (ref.)		1.00 (ref.)	
A2 30–299	606/24,908	5.6 (5.2–6.1)	4.1 (3.7–4.5)	1.48 (1.34–1.63)	<0.001	1.44 (1.31–1.58)	<0.001	1.33 (1.21–1.47)	<0.001
A3 ≥300	101/5130	5.1 (4.1–6.1)	2.8 (2.3–3.4)	1.44 (1.18–1.76)	<0.001	1.38 (1.13–1.69)	<0.001	1.25 (1.01–1.54)	<0.05
Lewy body dementia and Parkinson's disease with dementia	225/132,869	0.4 (0.3–0.4)	0.4 (0.3–0.4)						
A1 <30	188/102,831	0.4 (0.3–0.5)	0.4 (0.3–0.5)	1.00 (ref.)		1.00 (ref.)		1.00 (ref.)	
A2 30–299	33/24,908	0.3 (0.2–0.4)	0.2 (0.1–0.3)	0.73 (0.50–1.05)	0.09	0.68 (0.47–0.98)	<0.05	0.74 (0.51–1.09)	0.13
A3 ≥300	4/5130	0.2 (0.1–0.5)	0.1 (0.0–0.2)	0.50 (0.18–1.34)	0.17	0.44 (0.16–1.19)	0.11	0.47 (0.17–1.31)	0.15
Frontotemporal dementia	67/132,869	0.1 (0.1–0.1)	0.1 (0.1–0.1)						
A1 <30	50/102,831	0.1 (0.1–0.1)	0.1 (0.1–0.1)	1.00 (ref.)		1.00 (ref.)		1.00 (ref.)	
A2 30–299	13/24,908	0.1 (0.1–0.2)	0.1 (0.0–0.1)	1.08 (0.59–2.00)	0.80	1.06 (0.57–1.97)	0.85	1.16 (0.62–2.16)	0.65
A3 ≥300	4/5130	0.2 (0.1–0.5)	0.1 (0.0–0.2)	1.82 (0.66–5.06)	0.25	1.79 (0.64–4.99)	0.27	2.35 (0.81–6.82)	0.11
Unspecified dementia	2724/132,869	4.6 (4.4–4.8)	4.1 (4.0–4.3)						
A1 <30	1760/102,831	3.8 (3.6–4.0)	3.8 (3.6–4.0)	1.00 (ref.)		1.00 (ref.)		1.00 (ref.)	
A2 30–299	791/24,908	7.3 (6.8–7.9)	5.3 (4.9–5.7)	1.52 (1.40–1.66)	<0.001	1.53 (1.41–1.67)	<0.001	1.50 (1.37–1.63)	<0.001
A3 ≥300	173/5130	8.7 (7.4–10.0)	4.5 (3.8–5.2)	1.99 (1.71–2.33)	<0.001	2.01 (1.72–2.36)	<0.001	1.91 (1.62–2.25)	<0.001
Other dementia	220/132,869	0.4 (0.3–0.4)	0.3 (0.3–0.4)						
A1 <30	156/102,831	0.3 (0.3–0.4)	0.3 (0.3–0.4)	1.00 (ref.)		1.00 (ref.)		1.00 (ref.)	
A2 30–299	53/24,908	0.5 (0.4–0.6)	0.4 (0.3–0.5)	1.25 (0.91–1.71)	0.17	1.19 (0.87–1.64)	0.28	1.21 (0.88–1.67)	0.24
A3 ≥300	11/5130	0.6 (0.3–1.0)	0.3 (0.1–0.5)	1.51 (0.82–2.78)	0.19	1.40 (0.76–2.59)	0.28	1.36 (0.71–2.59)	0.36

*Note*: HRs and 95% confidence intervals were derived from Cox proportional hazards regression models. The 10‐year cumulative incidence was estimated using the Aalen–Johansen estimator, accounting for mortality as a competing event. Model 1: adjusted for age; model 2: model 1 + adjusted for sex, education, tobacco abuse, alcohol abuse, diagnosed obesity, cancer, depression, and hearing loss; model 3: model 2 + adjusted for diabetes, hypertension, congestive heart failure, myocardial infarction, atrial fibrillation, stroke, ACEi/ARBs, beta blockers, calcium channel blockers, NSAIDs, statins, and eGFR.

Abbreviations: ACEi, angiotensin‐converting enzyme inhibitor; ARB, angiotensin receptor blockers; eGFR, estimated glomerular filtration rate; HR, hazard ratio; IR, incidence rate; KDIGO, Kidney Disease Improving Global Outcomes; NSAIDs, nonsteroidal anti‐inflammatory agents; PY, person‐year.

We investigated the risk of all‐cause dementia incidence in subjects with different KDIGO ACR and eGFR categories (Table 3, Table S4). Within each eGFR stratum, a generally graded, higher risk of all‐cause dementia associated with higher ACR categories was observed, while not vice versa. No significant association between eGFR categories and the risk of all‐cause dementia was detected after a full adjustment, including baseline ACR.

**Table 3:** Adjusted hazard ratios (and 95% confidence intervals) for the incidence of all‐cause dementia by KDIGO combined albuminuria and eGFR categories.

eGFR, mL/min/1.73 m^2^	Albuminuria (mg/g)						Total	*p* value
	A1 <30	*p* value	A2 30–299	*p* value	A3 ≥300	*p* value		
G1 >90	1.00 (ref.)		1.31 (1.11–1.54)	<0.001	1.11 (0.71–1.74)	0.63	1.00 (ref.)	
G2 60–89	1.04 (0.96–1.13)	0.35	1.32 (1.20–1.45)	<0.001	1.39 (1.15–1.68)	<0.001	1.04 (0.97–1.12)	0.31
G3 30–59	0.99 (0.90–1.09)	0.87	1.21 (1.08–1.34)	<0.001	1.41 (1.19–1.66)	<0.001	0.98 (0.90–1.07)	0.68
G4 and G5 <30	1.04 (0.86–1.26)	0.68	1.24 (1.03–1.49)	<0.05	1.37 (1.10–1.71)	<0.001	1.01 (0.88–1.15)	0.90
Total	1.00 (ref.)		1.25 (1.19–1.31)	<0.001	1.37 (1.23–1.51)	<0.001		

*Note*: HRs and 95% confidence intervals were derived from Cox proportional hazards regression models. The “Total” column shows HRs for different eGFR categories, and the “Total” row shows HRs for different albuminuria categories. The remaining cells show HRs for different KDIGO combined albuminuria and eGFR categories. Models are adjusted for age, sex, education, tobacco abuse, alcohol abuse, diagnosed obesity, diabetes, hypertension, congestive heart failure, myocardial infarction, atrial fibrillation, stroke, cancer, hearing loss, depression, ACEi/ARBs, beta blockers, calcium channel blockers, NSAIDs, and statins.

Abbreviations: ACEi, angiotensin‐converting enzyme inhibitor; ARB, angiotensin receptor blockers; eGFR, estimated glomerular filtration rate; HR, hazard ratio; KDIGO, Kidney Disease Improving Global Outcomes; NSAIDs, nonsteroidal anti‐inflammatory agents.

### Albuminuria and the risk of type‐specific dementia incidence

HRs (95% CIs) for the associations between ACR and the risk of type‐specific dementia incidence are presented in Table 2. The 10‐year cumulative incidence of type‐specific dementia ranged from 0.1% (95% CI, 0.1%–0.1%) of developing FTD to 4.6% (95% CI, 4.4%–4.8%) of developing unspecified dementia. Compared with subjects with an ACR level of <30 mg/g, subjects with an ACR level of 30–299 mg/g had a 16% (HR, 1.16; 95% CI, 1.05–1.28), a 33% (HR, 1.33; 95% CI, 1.21–1.47), and a 50% (HR, 1.50; 95% CI, 1.37–1.63) higher risk of mixed AD, VaD, and unspecified dementia, respectively. Compared with subjects with an ACR level of <30 mg/g, subjects with an ACR level of ≥300 mg/g had a 25% (HR, 1.25; 95% CI, 1.01–1.54) and a 91% (HR, 1.91; 95% CI, 1.62–2.25) higher risk of VaD and unspecified dementia, respectively. For mixed AD, the association in subjects with an ACR level of ≥300 mg/g appeared suggestive but did not reach statistical significance (HR 1.19; 95% CI, 0.96–1.48). HRs for the incidence of AD, DLB, PDD, and FTD, as well as other types of dementia, were not significantly different in subjects with an ACR level of 30–299 and ≥300 mg/g versus subjects with an ACR level of <30 mg/g (all *p* > 0.05). When handled on a continuous scale, albuminuria generally showed similar results to those obtained using the ACR categories in a nonlinear fashion (*p*_nonlinearity_ < 0.001, Figs. S4 and S5).

### Sensitivity and supporting analyses

The results of sensitivity and supporting analyses generally corroborated the results of main analyses (Tables S5–S13). First, higher dipstick proteinuria categories were associated with a higher risk to develop all‐cause dementia, VaD, and unspecified dementia [HRs (95% CIs) for a dipstick proteinuria category of 1+ and 2+ versus negative, 1.15 (1.11–1.19), 1.19 (1.10–1.28), and 1.28 (1.20–1.36), respectively] in subjects tested for dipstick proteinuria (Tables S5 and S6). Second, the association of ACR categories with all‐cause dementia, VaD, and unspecified dementia was materially unchanged in the 1‐year landmark analysis, suggesting no evidence for the influences of reverse causation and detection bias on our results (Table S7). Third, in the analysis of negative control outcome, no significant associations between ACR categories and incident cataracts were observed, meaning no indication of unmeasured confounding that might distort to display the observed association between albuminuria and dementia (Table S8). Fourth, results remained largely unchanged when eGFR was calculated by the 2021 CKD‐EPI and the revised Lund–Malmo equations, indicating limited influences of using these eGFR equations on the observed association of albuminuria with dementia (Table S9). Fifth, when we redefined all‐cause dementia to include only AD, mixed AD, VaD, and unspecified dementia, the results were generally consistent, suggesting stable estimates of all‐cause dementia despite the heterogeneous effects of different dementia subtypes (Table S10). Sixth, the associations remained robust when we included subjects aged ≥50 years, indicating the limited impacts of modifying the age inclusion criteria on estimating the associations (Table S11). Seventh, when we started the study follow‐up from 2012 and reanalyzed the associations, results were essentially the same, implying that shifts in dementia case ascertainment due to changes in national coverage did not substantially affect the estimates (Table S12).

Additionally, in the subgroup analyses, we did not find evidence for possible effect modification by age, sex, hypertension, diabetes, and eGFR in the association between ACR and dementia (all *p*_interaction_ > 0.05, Fig. S6). In the competing risk analysis, although the magnitude of HRs for all‐cause dementia was attenuated, subjects with an ACR level of ≥30 mg/g robustly predicted a higher incidence of all‐cause dementia (subdistribution HR, 1.08; 95% CI, 1.03–1.13, Table S13).

## Discussion

In this large regional Swedish cohort study of persons undergoing albuminuria testing in the setting of universal healthcare, we found that increased levels of albuminuria were associated with a higher risk of all‐cause dementia, particularly mixed dementia, VaD, and unspecified dementia, independent of baseline eGFR. Importantly, the associations for all‐cause dementia, VaD, and unspecified dementia remained robust to several sensitivity and supporting analyses, including analyses replicated in individuals tested for dipstick proteinuria and handling mortality as a competing event.

Our key finding of a graded higher risk of all‐cause dementia incidence corroborates results from previous studies [30, 31, 32, 33, 34, 35, 36, 37]. For example, a Norwegian cohort that mainly comprised patients with self‐reported diabetes or hypertension reported that subjects with ACR in the highest cohort‐specific quantile had a 65% risk of developing all‐cause dementia when compared to subjects with ACR in the lowest quantile [31]. Similarly, in a population‐based US study, per 4.2‐fold increment in log‐transformed ACR was associated with a 27% higher risk of all‐cause dementia incidence [4]. Our findings also reinforce the previous evidence that the association between albuminuria and all‐dementia follows a nonlinear fashion [30, 32, 34, 37] and add to emphasize that this association remains robust regardless of the reference level of albuminuria being within (ACR of 10 mg/g) or above (ACR of 30 mg/g) the normal range. Moreover, our subgroup analyses of the association for all‐cause dementia reconcile the inconsistencies in previous findings about the role of age in the association for all‐cause dementia [4, 30, 33]. Together with these previous studies, our study strengthens the current evidence that the association between albuminuria and all‐cause dementia is consistent across different ranges of ages among older populations (≥65 years).

Another contribution of our study is that we tested the association of ACR across the different dementia subtypes. First, our results suggest that the risk of VaD, but not AD, increases across worsening albuminuria categories. This finding reconciles and expands on the previous evidence, which is still controversial about whether albuminuria is associated with both VaD and AD [30, 32, 34, 38] or not [33, 36, 37]. One possible explanation for these distinct results can lie in different study populations with divergent cultural backgrounds. Accumulating evidence supported variations in cognitive processes between individuals from “Eastern” and “Western” cultures (e.g., perceptual attention and reasoning) and the sequential influences of these variations on cognitive test performance [39]. Another explanation is that the pathways linking albuminuria to dementia can be partly type‐specific, and that type‐specific confounders are not sufficiently and distinctly addressed in previous studies. Thus far, possible mechanical pathways underlying the independent association between albuminuria and dementia are largely unknown, whereas the shared microvascular structure in the kidney and brain is the most frequently hypothesized mechanism [34, 40]. To be specific, cerebral vascular damage is highly involved in the development of VaD [7]. Differently, the deposition of Aβ peptide in the brain, following the inflammation‐induced damage to blood–brain barrier, is well‐established to play a key role in the development of AD [41]. As albuminuria is a marker reflecting endothelial damage and generalized vascular dysfunction [42], it is plausible to hypothesize that albuminuria captures pathogenic pathways more relevant to VaD than AD. This hypothesis is supported by our findings that a larger reduction in the magnitude of the association between albuminuria and VaD than that for AD after the adjustment for vascular risk factors (e.g., hypertension, diabetes, stroke). This hypothesis is also supported by our findings that the association of albuminuria with VaD is stronger than its association with mixed AD (combination of AD and VaD), and the association is even weaker with AD. Future studies are warranted to explore possible type‐specific mechanisms. Second, we also observe a notably higher risk of unspecified dementia in individuals with a higher level of albuminuria in this study. One possible explanation is that some patients are too frail to complete a full diagnostic assessment and thus receive a diagnosis of “unspecified dementia” while awaiting specialist evaluation [19]. This may reflect diagnostic uncertainty or misclassification in frailer populations, where complex and severe comorbidities could contribute to the inflated estimates.

To date, a few studies have investigated the independent and joint effects of albuminuria together with eGFR on estimating dementia risk [4, 34, 35, 43], whereas only one UK Biobank study and our study examined the dementia risk across a considerably expanded albuminuria and eGFR categorization according to the KDIGO guideline [13, 35], probably due to limited sample size in other previous studies. We found that a higher level of albuminuria is independently associated with a higher dementia risk within each eGFR stratum, while not vice versa, and that the association between eGFR and dementia was attenuated after adjustment for albuminuria. Additionally, our subgroup analyses showed that the association between albuminuria and dementia is consistently significant in subjects with preserved as well as impaired kidney function. We did not find the graded, higher dementia risk associated with lower eGFR categories within each albuminuria stratum as the other UKB study reported. Such inconsistency can be explained by the differences in the study population selected in our and the UK Biobank study. Contrary to the UK Biobank being population‐based, our registry‐based study included individuals indicated for ACR testing, who were generally older and had a higher proportion of diabetes. In the population represented by our study, the role of eGFR may be less significant due to more pronounced confounding effects on dementia. This contrasts with the robust role of albuminuria in dementia development, which appears to be consistent across different populations in our and the UK Biobank study. Different confounding effects on the role of eGFR on dementia due to distinct population selection may also explain why the association between eGFR and dementia is stronger in another SCREAM study than in this study [44]. In another SCREAM study, individuals were indicated for eGFR testing and had a comparably lower proportion of diabetes and hypertension.

Our real‐world data setting provides essential insights into possible bias relevant to observational studies when we interpret the association between albuminuria and dementia. First, our data advance our understanding of generalizability and possible selection bias related to different albuminuria testing pathways in estimating dementia risk. Dipstick proteinuria is mainly used for initial screening in primary care, whereas ACR is more accurate and indicated for further quantification of albumin among those who have a positive dipstick test or those at a high risk of developing increased albuminuria [13]. Although dipstick proteinuria is widely tested among elderly populations [45], prior research studying the association between dipstick proteinuria and dementia is limited and still has room for improvement in adjusting for eGFR and not positioning dipstick proteinuria only as a confounder [37, 46, 47]. The consistent results obtained from our separate analyses of persons tested for ACR and persons tested for dipstick proteinuria suggested the generalizability of the association between albuminuria and dementia across distinct clinical testing pathways. Furthermore, the limited influence of detection bias on the albuminuria dementia association is supported by the results of the 1‐year landmark analysis in our study, if we consider that subjects with albuminuria testing are more prone to receive workups that lead to dementia diagnoses. Second, the competing risk of mortality is expected to be substantially high in the investigation of the albuminuria dementia association, because albuminuria per se is a strong risk factor of mortality [48], and people receiving dementia diagnoses are predominantly aged populations with multiple comorbidities intrinsically at a high risk of mortality [49]. The unignorable competing risk of mortality is further supported by a 2‐fold higher incidence of mortality than that of all‐cause dementia, as well as a considerably strong cause‐specific risk of mortality documented in the current study. Our competing risk analyses showed that the cumulative incidence of dementia was slightly attenuated when accounting for mortality as a competing event, similar to results from another population‐based study [30]. Importantly, the magnitude and direction of associations remained largely consistent between the cause‐specific Cox and Fine and Gray models in both studies, thereby strengthening the evidence for a robust relationship between albuminuria and dementia from both etiologic and predictive perspectives. Notably, while the proportional hazards assumption was not met, the Fine and Gray models remain valid, with subdistribution HRs reflecting weighted averages of time‐varying effects over the follow‐up period [50, 51]. Third, as in all other observational studies, we cannot exclude possible bias due to residual confounding in the current study, such as diet, physical activity, socioeconomic status, or sex hormones [52], due to data availability constraints. Advanced methods such as instrumental variable analysis or trial emulation could help address unmeasured confounding and strengthen causal inference regarding the impact of albuminuria‐targeting interventions on dementia risk. Interestingly, previous Mendelian Randomization (MR) studies generally found no signals between genetically predicted albuminuria and dementia [53, 54]. It is noteworthy that MR studies could underestimate the associations due to technical restraints from verifying the absence of pleiotropy. From a perspective of triangulation, the prior and our studies together highlight the importance of employing other study designs in future investigation to strengthen causal inference linking albuminuria to dementia.

Notably, dementia is not yet curable, and preventing incident dementia by managing modifiable risk factors has received growing research interest. As there are several options for albuminuria‐lowering interventions (e.g., diet management of protein, glucose, and salt intake; cigarette cessation; sodium/glucose cotransporter 2 inhibitors; and ACEi/ARBs) [55], our findings provide pivotal implications for future studies to further investigate the adding value of albuminuria to contribute to the existing modifiable risk factor life‐course model of dementia prevention [3]. Our findings therefore indicate the need for future investigation, such as trial emulation, to examine how interventions like ACEIs or ARBs modify the risk of dementia among albuminuric patients [56]. Moreover, although our findings support an association between albuminuria and all‐cause dementia, further investigations are warranted to corroborate these results Montreal Cognitive Assessment, mini mental state examination (MMSE), domain‐specific cognitive tests, and instrumental kidney measurements, such as kidney imaging techniques. Furthermore, as our subgroup analyses showed that the association between albuminuria and dementia was consistently significant in subgroups by age and baseline eGFR, our findings also suggest that improved awareness and closer monitoring of dementia risk, particularly the risk of developing VaD, could be warranted among patients with increased levels of albuminuria, regardless of young or older age, and preserved or impaired kidney function. In addition, albuminuria is an early marker of kidney damage or diabetic nephropathy, as eGFR is typically normal or slightly elevated at the time when albuminuria has already increased [57]. Therefore, screening for albuminuria could enable earlier identification of individuals at risk for dementia.

The strength of our study includes a considerably large sample size, which allows us to investigate the associations for a broad range of dementia subtypes as well as the independent and joint effects of albuminuria with kidney function on estimating dementia risk. Furthermore, we ascertained dementia incidence via linkage to SveDem, of which the completeness and the reliability have been validated, especially in memory clinics [19, 58]. Our unique study setting of real‐world patients with universal free healthcare also mitigates the bias from distinct access to healthcare across different population subgroups [11]. Regarding limitations, we used clinical diagnoses to characterize lifestyle‐related phenotypes (e.g., diagnosed obesity, tobacco abuse) due to data availability in SCREAM. We recognize that the sensitivity of these clinical diagnoses can be low, whereas it is also plausible that the specificity of these clinical diagnoses is high. Besides, this study was based on a Swedish cohort, and extrapolation of our findings to other populations with different genetic, environmental, or healthcare backgrounds should therefore be cautious. Additionally, we did not conduct subgroup analyses stratified by baseline cognitive function [e.g., MMSE score] due to no data among our study population. Furthermore, the overall HR for all‐cause dementia may mask differences among subtypes. Therefore, it should be interpreted with caution, as it encompasses dementia types that may have distinct etiologies and associations with albuminuria. Lastly, as in all other registry‐based research, we cannot rule out possible selection bias, since selecting subjects based on their first‐available albuminuria test within the 2006–2019 window does not ensure that this reflects their first‐ever measurement or onset of albuminuria.

In conclusion, our statistical data suggest that increased albuminuria is associated with a higher risk of all‐cause dementia, particularly vascular and mixed dementia, independent of baseline eGFR and generalizable across individuals with distinct albuminuria testing pathways. These findings underscore the importance of routine albuminuria screening as part of early dementia risk assessment, particularly in patients with CKD or diabetic nephropathy. Early detection of albuminuria may enable more proactive management of kidney health and cognitive function, potentially delaying or preventing the onset of dementia.

## Author contributions

Li Luo, Ron T. Gansevoort, Juan‐Jesus Carrero, and Hong Xu conceived and designed the study. Juan‐Jesus Carrero and Maria Eriksdotter contributed to data acquisition. Li Luo conducted data analysis. All authors contributed to the interpretation of the data. Li Luo and Hong Xu drafted the manuscript. All authors revised the article. Ron T. Gansevoort, Juan‐Jesus Carrero, and Hong Xu supervised the work. All authors approved the final version of the manuscript.

## Conflict of interest statement

Dr. de Boer has received research grants and/or fees from AstraZeneca, Abbott, Boehringer Ingelheim, Cardior Pharmaceuticals GmbH, Novo Nordisk, and Roche; has had speaker engagements with and/or received fees from and/or served on an advisory board for Abbott, AstraZeneca, Bristol Myers Squibb, Cardior Pharmaceuticals GmbH, Novo Nordisk, and Roche; Dr. de Boer received travel support from Abbott, Cardior Pharmaceuticals GmbH, and Novo Nordisk. Other authors have none to declare for this article.

## Funding information

Li Luo is supported by a scholarship from the China Scholarship Council (CSC number: 202008440376). Dr. de Boer is supported by the European Research Council (ERC CoG 818715). Juan‐Jesus Carrero and the SCREAM project received support from the Swedish Research Council (2023‐01807), the US National Institutes of Health (NIH) (R01DK115534), the Swedish Heart and Lung Foundation (20230371), and Region Stockholm (ALF Medicine, FoUI‐986028). Hong Xu has received funding from StratNeuro grant (the Strategic Research Area Neuroscience‐Karolinska Institutet, Umeå University, and KTH), the Center for Innovative Medicine Foundation (CIMED, FoUI‐963369 and FoUI‐1002840), and the Swedish Research Council grant (#2022–01428).

## Supporting information




**Table S1**: ICD‐10 and ATC codes for identification of confounders and negative control outcomes.
**Table S2**: ICD‐10 and ATC codes for identification of dementia.
**Table S3**: Generalized variance‐inflation factors (GVIFs) to check multicollinearity among variables included in the fully adjusted model.
**Table S4**: The number of all‐cause dementia events and subjects, and incidence rate per 1000 person‐year according to the KDIGO combined albuminuria and eGFR categories. Each cell shows the number of events and the number of subjects at risk, as well as the incidence rate per 1000 person‐year (with 95% confidence interval between brackets).
**Table S5**: Baseline characteristics of the 152,480 subjects with available data on dipstick albuminuria, overall and stratified by dipstick albuminuria categories.
**Table S6**: Associations of dipstick albuminuria with the incidence of dementia, in the 152,480 subjects who had at least 1 dipstick albuminuria test.
**Table S7**: Associations of KDIGO albuminuria categories with the incidence of dementia using 1‐year landmark analysis.
**Table S8**: Associations of KDIGO albuminuria categories with the incidence of cataracts for the sensitivity analysis of negative control outcome.
**Table S9**: Associations of KDIGO albuminuria categories with the incidence of all‐cause dementia and type‐specific dementia, using the 2021 CKD‐EPI and the revised Lund‐Malmo equations to calculate eGFR.
**Table S10**: Associations of KDIGO albuminuria categories with the incidence of all‐cause dementia and type‐specific dementia, in the sensitivity analysis of defining all‐cause dementia as Alzheimer's disease, mixed dementia, vascular dementia and unspecified dementia.
**Table S11**: Associations of KDIGO albuminuria categories with the incidence of all‐cause dementia and type‐specific dementia, in the sensitivity analysis of including subjects aged ≥50 years.
**Table S12**: Associations of KDIGO albuminuria categories with the incidence of all‐cause dementia and type‐specific dementia, in the sensitivity analysis of starting the study follow‐up from 2012.
**Table S13**: Associations of KDIGO albuminuria categories with the incidence of dementia in the sensitivity analysis of handling mortality as a competing event.
**Figure S1**: Flow chart of study participants and study design.
**Figure S2**: Assessment of the proportional hazards assumption by the Schoenfeld residuals for the Cox model examining the risk of all‐cause dementia.
**Figure S3**: Adjusted hazard ratio of albuminuria with the incidence of all‐cause dementia (panel A) and distribution of albuminuria (panel B), with the reference standard for albuminuria being 30mg/g.
**Figure S4**: Adjusted hazard ratio of albuminuria with the incidence of type‐specific dementia.
**Figure S5**: Adjusted hazard ratio of albuminuria with the incidence of type‐specific dementia, with the reference standard for albuminuria being 30mg/g.
**Figure S6**: Subgroup analyses investigating effect modification of the association of KDIGO albuminuria categories (30‐299, and ≥300 versus <30 mg/g) with the incidence of dementia by age, sex, hypertension, diabetes, and eGFR.

**STROBE Statement**—Checklist of items that should be included in reports of *cohort studies*.

## Data Availability

The data contain patient‐related information and cannot be shared publicly as per European GDPR regulations. The data can be accessed through collaborative research applications addressed to the principal investigator Juan‐Jesus Carrero (juan.jesus.carrero@ki.se) and subjected to data sharing agreements that fulfill institutional and national regulations.
