# Supplementary material for: Albuminuria is associated with increased risk of dementia, independent of eGFR: The SCREAM project
Source: J Intern Med. 2025 Sep 23;298(5):489–503. doi: 10.1111/joim.70022 (PMC12522533; doi:10.1111/joim.70022)
Supplement: Supplementary file 1 — Table S1: ICD‐10 and ATC codes for identification of confounders and negative control outcomes. Table S2: ICD‐10 and ATC codes for identification of dementia. Table S3: Generalized variance‐inflation factors (GVIFs) to check multicollinearity among variables included in the fully adjusted model. Table S4: The number of all‐cause dementia events and subjects, and incidence rate per 1000 person‐year according to the KDIGO combined albuminuria and eGFR categories. Each cell shows the number of events and the number of subjects at risk, as well as the incidence rate per 1000 person‐year (with 95% confidence interval between brackets). Table S5: Baseline characteristics of the 152,480 subjects with available data on dipstick albuminuria, overall and stratified by dipstick albuminuria categories. Table S6: Associations of dipstick albuminuria with the incidence of dementia, in the 152,480 subjects who had at least 1 dipstick albuminuria test. Table S7: Associations of KDIGO albuminuria categories with the incidence of dementia using 1‐year landmark analysis. Table S8: Associations of KDIGO albuminuria categories with the incidence of cataracts for the sensitivity analysis of negative control outcome. Table S9: Associations of KDIGO albuminuria categories with the incidence of all‐cause dementia and type‐specific dementia, using the 2021 CKD‐EPI and the revised Lund‐Malmo equations to calculate eGFR. Table S10: Associations of KDIGO albuminuria categories with the incidence of all‐cause dementia and type‐specific dementia, in the sensitivity analysis of defining all‐cause dementia as Alzheimer's disease, mixed dementia, vascular dementia and unspecified dementia. Table S11: Associations of KDIGO albuminuria categories with the incidence of all‐cause dementia and type‐specific dementia, in the sensitivity analysis of including subjects aged ≥50 years. Table S12: Associations of KDIGO albuminuria categories with the incidence of all‐cause dementia and type‐specific dementia, [file JOIM-298-489-s001.pdf]

Table S1. ICD-10 and ATC codes for identification of confounders and negative control outcomes.

	ICD-10 codes	ATC codes
**Lifestyle factors**		
Tobacco abuse	F170, F171, F172, F179, Z716, Z720	
Alcohol abuse	E244, F10, G312, G621, G721,	
Obesity	E66	
**Comorbidities**		
Diabetes mellitus	E10-14	A10A, A10B
Hypertension	I10-15	C03, C07, C08, C09
Congestive heart failure	I099, I110, I130, I132, I255, I420, I425-429, I43, I50, P290	
Myocardial infarction	I21, I22, I252	
Atrial Fibrillation	I48	
Stroke	H341, I60, I61, I63, I64, I69	
Any Cancer (excluding non-melanoma skin cancer)	C00-C26, C30-C39, C40-C41, C43, C45-C49, C50-C58, C60-C76, C76-C80, C81-C96	
Depression	F204, F32, F33	N06A
Hearing loss	H833, H90, H91	
**Medications**		
ACEi/ARBs		C09
Beta blockers		C07
Calcium channel blockers		C08
NSAIDS		B01AC06, N02BA01, M01A
Statins		C10AA, C10B
**Negative control outcomes**		
Psoriasis	L40	
Cataracts	H25, H26	
Disorders of thyroid gland	E00, E01, E02, E03, E04, E05, E06, E07	

Abbreviations: ACEi, angiotensin-converting enzyme inhibitor; ARB, angiotensin receptor blockers; NSAIDs, nonsteroidal anti-inflammatory agent.

Table S2. ICD-10 and ATC codes for identification of dementia.

	ICD-10 codes	ATC codes
All-cause dementia	F00, F01, F02, F03, F051, G30, G31	N06DA02, N06DA03<br>N06DA04, N06DX01
Type-specific dementia		
Alzheimer's disease	F001, G301	
Mixed dementia	F002, G308	
Vascular dementia	F01	
Lewy Body dementia and Parkinson's	G318, F023	
Frontotemporal dementia	F020, G310	
Unspecified dementia	F03, G319	
** Other dementia	F021, F022, F024, F028, F051, G311, G312	

** Other miscellaneous types, such as cortico-basal syndrome or alcohol-related dementia.

Table S3. Generalized variance-inflation factors (GVIFs) to check multicollinearity among variables included in the fully adjusted model.

	GVIF	Df	GVIF^(1/(2*Df))
KDIGO albuminuria categories (mg/g)	1.19	2	1.05
Age, years	1.40	1	1.18
Female	1.08	1	1.04
Highest attained education	1.05	2	1.01
Tobacco abuse	1.02	1	1.01
Alcohol abuse	1.04	1	1.02
Diagnosed obesity	1.04	1	1.02
Diabetes mellitus	1.10	1	1.05
Hypertension	1.63	1	1.28
Congestive heart failure	1.36	1	1.17
Myocardial infarction	1.25	1	1.12
Atrial fibrillation	1.30	1	1.14
Stroke	1.06	1	1.03
Cancer	1.01	1	1.01
Depression	1.03	1	1.02
Hearing loss	1.03	1	1.01
ACEi/ARBs	1.39	1	1.18
Beta blockers	1.38	1	1.17
Calcium channel blocker	1.12	1	1.06
NSAIDs	1.15	1	1.07
Statins	1.21	1	1.10
eGFR category, ml/min/1.73 m^2	1.43	3	1.06

The fully adjusted model examines the association between KDIGO albuminuria categories and all-cause dementia, in the 132,869 subjects who had at least 1 ACR test.

The fully adjusted model is adjusted for age, sex, education, tobacco abuse, alcohol abuse, diagnosed obesity, cancer, depression, hearing loss, diabetes, hypertension, congestive heart failure, myocardial infarction, atrial fibrillation, stroke, ACEi/ARBs, beta blockers, calcium channel blockers, NSAIDs, statins, and eGFR.

Abbreviations: ACEi, angiotensin-converting enzyme inhibitor; ACR, urine albumin-creatinine ratio; ARB, angiotensin receptor blockers; Df, degrees of freedom; eGFR, estimated glomerular filtration rate; GVIF, generalized variance-inflation factor; KDIGO, Kidney Disease Improving Global Outcomes; NSAIDs, nonsteroidal anti-inflammatory agents.

Table S4. The number of all-cause dementia events and subjects, and incidence rate per 1000 person-year according to the KDIGO combined albuminuria and eGFR categories. Each cell shows the number of events and the number of subjects at risk, as well as the incidence rate per 1000 person-year (with 95% confidence interval between brackets).

eGFR, ml/min/1.73 m^2	Albuminuria, mg/g		
	A1<br><30	A2<br>30-299	A3<br>≥300
G1 >90	704/16,452<br>8.4 (7.8- 9.1)	175/3,031<br>11.6 (10.0-13.5)	20/350<br>11.4 (6.9-17.5)
G2 60-89	4,218/65,660<br>14.4 (13.9-14.8)	1,224/12,367<br>21.8 (20.6-23.1)	129/1,450<br>20.9 (17.5-24.9)
G3 30-59	1,612/19,419<br>19.8 (18.8-20.8)	796/7,598<br>25.9 (24.1-27.8)	189/1,897<br>24.5 (21.1-28.2)
G4 & G5 <30	129/1,300<br>26.1 (21.8-31.0)	146/1,912<br>24.6 (20.8-28.9)	93/1,433<br>21.4 (17.3-26.2)

Abbreviations: eGFR, estimated glomerular filtration rate; KDIGO, Kidney Disease Improving Global Outcomes.

Table S5. Baseline characteristics of the 152,480 subjects with available data on dipstick albuminuria, overall and stratified by dipstick albuminuria categories.

	Overall	Dipstick albuminuria categories		
		negative	1+	≥2+
No. of subjects	152,480	118,873	19,049	14,558
**Demographics**				
Age, years	74.0 (69.0, 80.0)	73.0 (69.0, 80.0)	75.0 (70.0, 82.0)	76.0 (70.0, 83.0)
Age category, years				
<70	44,038 (28.9%)	36,042 (30.3%)	4,660 (24.5%)	3,336 (22.9%)
70-75	36,987 (24.3%)	29,493 (24.8%)	4,247 (22.3%)	3,247 (22.3%)
75-80	29,162 (19.1%)	22,623 (19.0%)	3,756 (19.7%)	2,783 (19.1%)
80-85	21,756 (14.3%)	16,346 (13.8%)	2,992 (15.7%)	2,418 (16.6%)
≥85	20,537 (13.5%)	14,369 (12.1%)	3,394 (17.8%)	2,774 (19.1%)
Female	82,226 (53.9%)	67,799 (57.0%)	8,818 (46.3%)	5,609 (38.5%)
Highest attained education				
Compulsory school	46,097 (30.2%)	35,087 (29.5%)	6,122 (32.1%)	4,888 (33.6%)
Secondary school	60,974 (40.0%)	47,373 (39.9%)	7,594 (39.9%)	6,007 (41.3%)
University	45,409 (29.8%)	36,413 (30.6%)	5,333 (28.0%)	3,663 (25.2%)
**Lifestyle**				
Tobacco abuse	2,078 (1.4%)	1,381 (1.2%)	352 (1.8%)	345 (2.4%)
Alcohol abuse	5,119 (3.4%)	3,625 (3.0%)	819 (4.3%)	675 (4.6%)
Diagnosed obesity	7,960 (5.2%)	5,921 (5.0%)	1,110 (5.8%)	929 (6.4%)
**Kidney function**				
eGFR, ml/min/1.73 m^2	74.2 (59.5, 85.8)	75.6 (61.8, 86.4)	70.9 (54.5, 84.1)	64.6 (45.7, 81.2)
eGFR category, ml/min/1.73 m^2				
≥90	21,816 (14.3%)	18,049 (15.2%)	2,343 (12.3%)	1,424 (9.8%)
60-89	91,457 (60.0%)	74,048 (62.3%)	10,566 (55.5%)	6,843 (47.0%)
30-59	33,873 (22.2%)	24,199 (20.4%)	5,057 (26.5%)	4,617 (31.7%)
<30	5,334 (3.5%)	2,577 (2.2%)	1,083 (5.7%)	1,674 (11.5%)
**Comorbidities**				
Diabetes mellitus	29,290 (19.2%)	21,268 (17.9%)	4,069 (21.4%)	3,953 (27.2%)
Hypertension	106,688 (70.0%)	81,243 (68.3%)	13,952 (73.2%)	11,493 (78.9%)
Congestive heart failure	18,329 (12.0%)	12,712 (10.7%)	2,876 (15.1%)	2,741 (18.8%)
Myocardial infarction	13,385 (8.8%)	9,462 (8.0%)	1,952 (10.2%)	1,971 (13.5%)
Atrial fibrillation	22,211 (14.6%)	15,451 (13.0%)	3,545 (18.6%)	3,215 (22.1%)
Stroke	13,270 (8.7%)	9,250 (7.8%)	2,075 (10.9%)	1,945 (13.4%)
Cancer	35,678 (23.4%)	26,383 (22.2%)	5,075 (26.6%)	4,220 (29.0%)
Depression	14,636 (9.6%)	11,379 (9.6%)	1,924 (10.1%)	1,333 (9.2%)
Hearing loss	19,991 (13.1%)	15,187 (12.8%)	2,709 (14.2%)	2,095 (14.4%)
**Medication**				
ACEi/ARBs	61,717 (40.5%)	46,717 (39.3%)	7,940 (41.7%)	7,060 (48.5%)
Beta blockers	55,109 (36.1%)	41,096 (34.6%)	7,443 (39.1%)	6,570 (45.1%)

Calcium channel blocker	34,577 (22.7%)	25,383 (21.4%)	4,675 (24.5%)	4,519 (31.0%)
NSAIDs	63,786 (41.8%)	49,145 (41.3%)	8,317 (43.7%)	6,324 (43.4%)
Statins	45,555 (29.9%)	34,826 (29.3%)	5,762 (30.2%)	4,967 (34.1%)

Continuous variables are reported as median (interquartile range) and categorical variables are reported as N (%). Abbreviations: ACEi, angiotensin-converting enzyme inhibitor; ARB, angiotensin receptor blockers; eGFR, estimated glomerular filtration rate; NSAIDs, nonsteroidal anti-inflammatory agent.

Table S6. Associations of dipstick albuminuria with the incidence of dementia, in the 152,480 subjects who had at least 1 dipstick albuminuria test.

Dipstick albuminuria categories	No. of events/participants	IR per 1000PY (95% CI)	10-year cumulative incidence (%, 95%CI)	Model 1, HR (95%CI)	P value	Model 2, HR (95%CI)	P value
**All-cause dementia**	17,448/152,480	20.8 (20.5-21.1)	15.3 (15.1-15.6)				
negative	13,630/118,873	19.8 (19.5-20.2)	15.3 (15.0-15.5)	1.00 (ref)		1.00 (ref)	
1+	2,370/19,049	25.9 (24.9-27.0)	16.7 (16.0-17.4)	1.18 (1.13-1.24)	<0.001	1.18 (1.13-1.24)	<0.001
≥2+	1,448/14,558	24.1 (22.9-25.4)	14.1 (13.3-14.8)	1.08 (1.03-1.14)	<0.001	1.10 (1.04-1.16)	<0.001
1+ & 2+	3,818/33,607	25.2 (24.4-26.0)	15.3 (15.0-15.5)	1.14 (1.10-1.19)	<0.001	1.15 (1.11-1.19)	<0.001
**Type-specific dementia**							
**Alzheimer's disease**	3,565/152,480	4.2 (4.1-4.4)	3.3 (3.2-3.4)				
negative	2,931/118,873	4.3 (4.1-4.4)	3.4 (3.3-3.6)	1.00 (ref)		1.00 (ref)	
1+	431/19,049	4.7 (4.3-5.2)	3.3 (2.9-3.6)	1.02 (0.92-1.13)	0.72	1.12 (1.01-1.24)	<0.05
≥2+	203/14,558	3.4 (2.9-3.9)	2.1 (1.8-2.4)	0.72 (0.62-0.83)	<0.001	0.87 (0.75-1.01)	0.06
1+ & 2+	634/33,607	4.2 (3.9-4.5)	3.4 (3.3-3.6)	0.90 (0.82-0.98)	<0.05	1.03 (0.94-1.12)	0.56
**Mixed dementia**	3,948/152,480	4.7 (4.6-4.9)	3.8 (3.6-3.9)				
negative	3,121/118,873	4.5 (4.4-4.7)	3.8 (3.6-3.9)	1.00 (ref)		1.00 (ref)	
1+	523/19,049	5.7 (5.2-6.2)	4.1 (3.7-4.4)	1.15 (1.05-1.26)	<0.001	1.16 (1.06-1.27)	<0.001
≥2+	304/14,558	5.1 (4.5-5.7)	3.2 (2.8-3.6)	1.01 (0.90-1.14)	0.88	1.02 (0.91-1.15)	0.74
1+ & 2+	827/33,607	5.5 (5.1-5.8)	3.8 (3.6-3.9)	1.10 (1.01-1.18)	<0.05	1.11 (1.02-1.20)	<0.05
**Vascular dementia**	3,573/152,480	4.3 (4.1-4.4)	3.4 (3.3-3.5)				
negative	2,719/118,873	4.0 (3.8-4.1)	3.3 (3.2-3.5)	1.00 (ref)		1.00 (ref)	
1+	523/19,049	5.7 (5.2-6.2)	4.0 (3.6-4.3)	1.31 (1.19-1.44)	<0.001	1.23 (1.12-1.35)	<0.001
≥2+	331/14,558	5.5 (4.9-6.1)	3.6 (3.1-4.0)	1.25 (1.11-1.40)	<0.001	1.12 (1.00-1.26)	0.06
1+ & 2+	854/33,607	5.6 (5.3-6.0)	3.3 (3.2-3.5)	1.29 (1.19-1.39)	<0.001	1.19 (1.10-1.28)	<0.001
**Lewy Body dementia and Parkinson's Disease with dementia**	492/152,480	0.6 (0.5-0.6)	0.5 (0.4-0.5)				
negative	401/118,873	0.6 (0.5-0.6)	0.5 (0.4-0.5)	1.00 (ref)		1.00 (ref)	
1+	55/19,049	0.6 (0.5-0.8)	0.4 (0.3-0.6)	1.02 (0.77-1.35)	0.92	0.97 (0.73-1.29)	0.85
≥2+	36/14,558	0.6 (0.4-0.8)	0.3 (0.2-0.5)	1.01 (0.72-1.43)	0.93	1.00 (0.71-1.42)	1,00
1+ & 2+	91/33,607	0.6 (0.5-0.7)	0.5 (0.4-0.5)	1.01 (0.81-1.28)	0.90	0.98 (0.78-1.24)	0.89
**Frontotemporal dementia**	121/152,480	0.1 (0.1-0.2)	0.1 (0.1-0.1)				
negative	106/118,873	0.2 (0.1-0.2)	0.1 (0.1-0.2)	1.00 (ref)		1.00 (ref)	
1+	7/19,049	0.1 (0.0-0.2)	0.1 (0.0-0.1)	0.50 (0.23-1.07)	0.07	0.49 (0.23-1.06)	0.07
≥2+	8/14,558	0.1 (0.1-0.3)	0.1 (0.0-0.1)	0.86 (0.42-1.76)	0.68	0.88 (0.42-1.83)	0.74
1+ & 2+	15/33,607	0.1 (0.1-0.2)	0.1 (0.1-0.2)	0.64 (0.37-1.10)	0.11	0.64 (0.37-1.11)	0.11

**Unspecified dementia**	5,335/152,480	6.4 (6.2-6.5)	5.0 (4.9-5.2)				
negative	4,025/118,873	5.9 (5.7-6.0)	4.8 (4.7-5.0)	1.00 (ref)		1.00 (ref)	
1+	779/19,049	8.5 (7.9-9.1)	5.9 (5.5-6.4)	1.28 (1.19-1.38)	<0.001	1.26 (1.17-1.37)	<0.001
≥2+	531/14,558	8.9 (8.1-9.6)	5.6 (5.1-6.1)	1.31 (1.20-1.44)	<0.001	1.30 (1.19-1.43)	<0.001
1+ & 2+	1,310/33,607	8.7 (8.2-9.1)	4.8 (4.7-5.0)	1.29 (1.21-1.38)	<0.001	1.28 (1.20-1.36)	<0.001
**Other dementia**	414/152,480	0.5 (0.4-0.5)	0.4 (0.4-0.5)				
negative	327/118,873	0.5 (0.4-0.5)	0.4 (0.4-0.5)	1.00 (ref)		1.00 (ref)	
1+	52/19,049	0.6 (0.4-0.7)	0.5 (0.3-0.6)	1.10 (0.82-1.47)	0.54	1.04 (0.77-1.39)	0.81
≥2+	35/14,558	0.6 (0.4-0.8)	0.4 (0.2-0.5)	1.11 (0.78-1.58)	0.55	1.04 (0.73-1.48)	0.83
1+ & 2+	87/33,607	0.6 (0.5-0.7)	0.4 (0.4-0.5)	1.10 (0.87-1.40)	0.42	1.04 (0.82-1.32)	0.76

HRs and 95% confidence intervals were derived from Cox proportional hazards regression models.

The 10-year cumulative incidence was estimated using the Aalen-Johansen estimator, accounting for mortality as a competing event.

Model 1: adjusted for age;

model 2: model 1 + adjusted for sex, education, tobacco abuse, alcohol abuse, diagnosed obesity, cancer, depression, hearing loss, diabetes, hypertension, congestive heart failure, myocardial infarction, atrial fibrillation, stroke, ACEi/ARBs, beta blockers, calcium channel blockers, NSAIDs, statins, and eGFR.

Abbreviations: ACEi, angiotensin-converting enzyme inhibitor; ARB, angiotensin receptor blockers; eGFR, estimated glomerular filtration rate; HR, hazard ratio; IR, incidence rate; KDIGO, Kidney Disease Improving Global Outcomes; NSAIDs, nonsteroidal anti-inflammatory agents; PY, person-year.

Table S7. Associations of KDIGO albuminuria categories with the incidence of dementia using 1-year landmark analysis.

KDIGO albuminuria categories, mg/g	No. of events/participants	HR (95%CI)	P value
**All-cause dementia**	8,031/112,959		
A1 <30	5,798/88,065	1.00 (ref)	
A2 30-299	1,900/20,839	1.20 (1.14- 1.26)	<0.001
A3 ≥300	333/4,055	1.29 (1.15- 1.44)	<0.001
**Type-specific dementia**			
**Alzheimer's disease**	1,430/112,975		
A1 <30	1,149/88,075	1.00 (ref)	
A2 30-299	249/20,844	0.98 (0.86- 1.13)	0.82
A3 ≥300	32/4,056	0.88 (0.62- 1.27)	0.50
**Mixed dementia**	1,969/112,975		
A1 <30	1,456/88,075	1.00 (ref)	
A2 30-299	437/20,844	1.10 (0.98- 1.22)	0.10
A3 ≥300	76/4,056	1.18 (0.93- 1.50)	0.18
**Vascular dementia**	1,870/112,975		
A1 <30	1,291/88,075	1.00 (ref)	
A2 30-299	498/20,844	1.28 (1.15- 1.43)	<0.001
A3 ≥300	81/4,056	1.20 (0.95- 1.51)	0.12
**Lewy Body dementia and Parkinson's Disease with dementia**	200/112,975		
A1 <30	171/88,075	1.00 (ref)	
A2 30-299	27/20,844	0.71 (0.47- 1.07)	0.10
A3 ≥300	2/4,056	0.32 (0.08- 1.31)	0.11
**Frontotemporal dementia**	57/112,975		
A1 <30	43/88,075	1.00 (ref)	
A2 30-299	12/20,844	1.21 (0.63- 2.34)	0.57
A3 ≥300	2/4,056	1.28 (0.30- 5.52)	0.74
**Unspecified dementia**	2,336/112,975		
A1 <30	1,564/88,075	1.00 (ref)	
A2 30-299	640/20,844	1.40 (1.27- 1.54)	<0.001
A3 ≥300	132/4,056	1.75 (1.45- 2.11)	<0.001
**Other dementia**	185/112,975		
A1 <30	134/88,075	1.00 (ref)	
A2 30-299	42/20,844	1.16 (0.81- 1.65)	0.43
A3 ≥300	9/4,056	1.42 (0.70- 2.89)	0.33

HRs and 95% confidence intervals were derived from Cox proportional hazards regression models.

Models are adjusted for age, sex, education, tobacco abuse, alcohol abuse, diagnosed obesity, diabetes, hypertension, congestive heart failure, myocardial infarction, atrial fibrillation, stroke, cancer, depression, hearing loss, ACEi/ARBs, beta blockers, calcium channel blockers, NSAIDs, statins, and eGFR.

Abbreviations: ACEi, angiotensin-converting enzyme inhibitor; ARB, angiotensin receptor blockers; eGFR, estimated glomerular filtration rate; HR, hazard ratio; KDIGO, Kidney Disease Improving Global Outcomes; NSAIDs, nonsteroidal anti-inflammatory agents.

Table S8. Associations of KDIGO albuminuria categories with the incidence of cataracts for the sensitivity analysis of negative control outcome.

KDIGO albuminuria categories, mg/g	No. of events/participants	IR per 1000PY (95% CI)	Model 1, HR (95%CI)	Model 2, HR (95%CI)
**Cataracts**	32,021/100,884	80.7 (79.8-81.6)		
A1 <30	25,124/78,154	80.7 (79.7-81.7)	1.00 (ref)	1.00 (ref)
A2 30-299	5,870/18,785	81.5 (79.4-83.6)	0.98 (0.96-1.01)	1.00 (0.97-1.03)
A3 ≥300	1,027/3,945	75.4 (70.9-80.2)	0.93 (0.87-0.99)	0.96 (0.90-1.03)

HRs and 95% confidence intervals were derived from Cox proportional hazards regression models.

Model 1: adjusted for age;

Model 2: adjusted for sex, education, tobacco abuse, alcohol abuse, diagnosed obesity, cancer, depression, hearing loss, diabetes, hypertension, congestive heart failure, myocardial infarction, atrial fibrillation, stroke, ACEi/ARBs, beta blockers, calcium channel blockers, NSAIDs, statins, and eGFR.

Abbreviations: ACEi, angiotensin-converting enzyme inhibitor; ARB, angiotensin receptor blockers; eGFR, estimated glomerular filtration rate; HR, hazard ratio; IR, incidence rate; KDIGO, Kidney Disease Improving Global Outcomes; NSAIDs, nonsteroidal anti-inflammatory agents; PY, person-year.

Table S9. Associations of KDIGO albuminuria categories with the incidence of all-cause dementia and type-specific dementia, using the 2021 CKD-EPI and the revised Lund-Malmo equations to calculate eGFR.

KDIGO albuminuria categories, mg/g	No. of events/participants	HR (95%CI), using the 2009 CKD-EPI equation	P value	HR (95%CI), using the 2021 CKD-EPI equation	P value	HR (95%CI), using the revised Lund-Malmo equation	P value
**All-cause dementia**	9,435/132,869						
A1 <30	6,663/102,831	1.00 (ref)		1.00 (ref)		1.00 (ref)	
A2 30-299	2,341/24,908	1.25 (1.19-1.31)	<0.001	1.25 (1.19-1.31)	<0.001	1.25 (1.19-1.31)	<0.001
A3 ≥300	431/5,130	1.37 (1.23-1.51)	<0.001	1.37 (1.24-1.52)	<0.001	1.37 (1.24-1.51)	<0.001
**Type-specific dementia**							
**Alzheimer’s disease**	1,725/132,869						
A1 <30	1,376/102,831	1.00 (ref)		1.00 (ref)		1.00 (ref)	
A2 30-299	303/24,908	0.97 (0.85-1.10)	0.61	0.97 (0.85-1.10)	0.65	0.97 (0.85-1.10)	0.65
A3 ≥300	46/5,130	0.99 (0.74-1.34)	0.97	1.02 (0.76-1.38)	0.89	1.01 (0.74-1.36)	0.97
**Mixed dementia**	2,293/132,869						
A1 <30	1,659/102,831	1.00 (ref)		1.00 (ref)		1.00 (ref)	
A2 30-299	542/24,908	1.16 (1.05-1.28)	<0.001	1.16 (1.05-1.28)	<0.001	1.16 (1.05-1.28)	<0.001
A3 ≥300	92/5,130	1.19 (0.96-1.48)	0.12	1.21 (0.97-1.50)	0.09	1.20 (0.97-1.49)	0.10
**Vascular dementia**	2,181/132,869						
A1 <30	1,474/102,831	1.00 (ref)		1.00 (ref)		1.00 (ref)	
A2 30-299	606/24,908	1.33 (1.21-1.47)	<0.001	1.33 (1.20-1.46)	<0.001	1.32 (1.20-1.46)	<0.001
A3 ≥300	101/5,130	1.25 (1.01-1.54)	<0.05	1.23 (0.99-1.51)	0.06	1.23 (1.00-1.52)	0.05
**Lewy Body dementia and Parkinson's Disease with dementia**	225/132,869						
A1 <30	188/102,831	1.00 (ref)		1.00 (ref)		1.00 (ref)	
A2 30-299	33/24,908	0.74 (0.51-1.09)	0.13	0.73 (0.50-1.07)	0.11	0.72 (0.49-1.06)	0.10
A3 ≥300	4/5,130	0.47 (0.17-1.31)	0.15	0.44 (0.16-1.24)	0.12	0.45 (0.16-1.24)	0.12
**Frontotemporal dementia**	67/132,869						
A1 <30	50/102,831	1.00 (ref)		1.00 (ref)		1.00 (ref)	
A2 30-299	13/24,908	1.16 (0.62-2.16)	0.65	1.14 (0.61-2.14)	0.68	1.13 (0.60-2.12)	0.71
A3 ≥300	4/5,130	2.35 (0.81-6.82)	0.11	2.23 (0.77-6.52)	0.14	2.21 (0.76-6.42)	0.15
**Unspecified dementia**	2,724/132,869						
A1 <30	1,760/102,831	1.00 (ref)		1.00 (ref)		1.00 (ref)	
A2 30-299	791/24,908	1.50 (1.37-1.63)	<0.001	1.50 (1.38-1.64)	<0.001	1.49 (1.37-1.63)	<0.001
A3 ≥300	173/5,130	1.91 (1.62-2.25)	<0.001	1.93 (1.64-2.27)	<0.001	1.93 (1.64-2.27)	<0.001
**Other dementia**	220/132,869						
A1 <30	156/102,831	1.00 (ref)		1.00 (ref)		1.00 (ref)	

A2 30-299	53/24,908	1.21 (0.88-1.67)	0.24	1.22 (0.89-1.69)	0.22	1.19 (0.86-1.64)	0.29
A3 ≥300	11/5,130	1.36 (0.71-2.59)	0.36	1.39 (0.73-2.67)	0.32	1.30 (0.68-2.46)	0.43

HRs and 95% confidence intervals were derived from Cox proportional hazards regression models.

Models are adjusted for age, sex, education, tobacco abuse, alcohol abuse, diagnosed obesity, cancer, depression, hearing loss, diabetes, hypertension, congestive heart failure, myocardial infarction, atrial fibrillation, stroke, ACEi/ARBs, beta blockers, calcium channel blockers, NSAIDs, statins, and eGFR.

Abbreviations: ACEi, angiotensin-converting enzyme inhibitor; ACR, urine albumin-creatinine ratio; ARB, angiotensin receptor blockers; eGFR, estimated glomerular filtration rate; HR, hazard ratio; KDIGO, Kidney Disease Improving Global Outcomes; NSAIDs, nonsteroidal anti-inflammatory agents.

Table S10. Associations of KDIGO albuminuria categories with the incidence of all-cause dementia and type-specific dementia, in the sensitivity analysis of defining all-cause dementia as Alzheimer's disease, mixed dementia, vascular dementia and unspecified dementia.

KDIGO albuminuria categories, mg/g	No. of events/participants	IR per 1000PY (95% CI)	10-year cumulative incidence (%, 95%CI)	Model 1, HR (95%CI)	P value	Model 2, HR (95%CI)	P value	Model 3, HR (95%CI)	P value
**All-cause dementia**	8,963/133,244	15.1 (14.8-15.4)	12.7 (12.4-13.0)						
A1 <30	6,320/103,087	13.6 (13.3-13.9)	12.5 (12.1-12.8)	1.00 (ref)		1.00 (ref)		1.00 (ref)	
A2 30-299	2,235/25,008	20.6 (19.8-21.5)	14.0 (13.4-14.6)	1.25 (1.19-1.31)	<0.001	1.26 (1.20-1.32)	<0.001	1.24 (1.18-1.31)	<0.001
A3 ≥300	408/5,149	20.3 (18.4-22.4)	10.3 (9.3-11.3)	1.34 (1.21-1.48)	<0.001	1.35 (1.22-1.49)	<0.001	1.35 (1.22-1.50)	<0.001
**Type-specific dementia**									
**Alzheimer's disease**	1,772/133,244	3.0 (2.8-3.1)	2.6 (2.5-2.7)						
A1 <30	1,413/103,087	3.0 (2.9-3.2)	2.8 (2.6-3.0)	1.00 (ref)		1.00 (ref)		1.00 (ref)	
A2 30-299	312/25,008	2.9 (2.6-3.2)	2.2 (1.9-2.4)	0.81 (0.72-0.92)	<0.001	0.87 (0.77-0.98)	<0.05	0.96 (0.85-1.09)	0.53
A3 ≥300	47/5,149	2.3 (1.7-3.1)	1.3 (0.9-1.7)	0.70 (0.53-0.94)	<0.05	0.78 (0.58-1.05)	0.10	0.98 (0.72-1.32)	0.88
**Mixed dementia**	2,337/133,244	3.9 (3.8-4.1)	3.5 (3.4-3.7)						
A1 <30	1,692/103,087	3.6 (3.5-3.8)	3.6 (3.4-3.8)	1.00 (ref)		1.00 (ref)		1.00 (ref)	
A2 30-299	553/25,008	5.1 (4.7-5.5)	3.7 (3.4-4.1)	1.18 (1.07-1.30)	<0.001	1.17 (1.06-1.29)	<0.001	1.16 (1.05-1.28)	<0.001
A3 ≥300	92/5,149	4.6 (3.7-5.6)	2.5 (2.0-3.1)	1.14 (0.92-1.41)	0.22	1.13 (0.92-1.40)	0.25	1.16 (0.93-1.44)	0.18
**Vascular dementia**	2,231/133,244	3.8 (3.6-3.9)	3.4 (3.2-3.6)						
A1 <30	1,510/103,087	3.2 (3.1-3.4)	3.2 (3.0-3.4)	1.00 (ref)		1.00 (ref)		1.00 (ref)	
A2 30-299	619/25,008	5.7 (5.3-6.2)	4.2 (3.8-4.5)	1.47 (1.34-1.62)	<0.001	1.43 (1.30-1.58)	<0.001	1.33 (1.21-1.46)	<0.001
A3 ≥300	102/5,149	5.1 (4.1-6.2)	2.8 (2.3-3.4)	1.42 (1.16-1.73)	<0.001	1.36 (1.11-1.66)	<0.001	1.24 (1.01-1.53)	<0.05
**Unspecified dementia**	2,623/133,244	4.4 (4.3-4.6)	3.9 (3.8-4.1)						
A1 <30	1,705/103,087	3.7 (3.5-3.8)	3.6 (3.4-3.8)	1.00 (ref)		1.00 (ref)		1.00 (ref)	
A2 30-299	751/25,008	6.9 (6.4-7.4)	4.9 (4.5-5.3)	1.48 (1.36-1.62)	<0.001	1.48 (1.36-1.62)	<0.001	1.45 (1.32-1.58)	<0.001
A3 ≥300	167/5,149	8.3 (7.1-9.7)	4.2 (3.6-4.9)	1.97 (1.68-2.31)	<0.001	1.98 (1.68-2.32)	<0.001	1.87 (1.58-2.21)	<0.001

HRs and 95% confidence intervals were derived from Cox proportional hazards regression models.

The 10-year cumulative incidence was estimated using the Aalen-Johansen estimator, accounting for mortality as a competing event.

Model 1: adjusted for age;

model 2: model 1 + adjusted for sex, education, tobacco abuse, alcohol abuse, diagnosed obesity, cancer, depression, and hearing loss;

model 3: model 2 + adjusted for diabetes, hypertension, congestive heart failure, myocardial infarction, atrial fibrillation, stroke, ACEi/ARBs, beta blockers, calcium channel blockers, NSAIDs, statins, and eGFR.

Abbreviations: ACEi, angiotensin-converting enzyme inhibitor; ARB, angiotensin receptor blockers; eGFR, estimated glomerular filtration rate; HR, hazard ratio; IR, incidence rate; KDIGO, Kidney Disease Improving Global Outcomes; NSAIDs, nonsteroidal anti-inflammatory agents; PY, person-year.

Table S11. Associations of KDIGO albuminuria categories with the incidence of all-cause dementia and type-specific dementia, in the sensitivity analysis of including subjects aged ⩾50 years.

KDIGO albuminuria categories, mg/g	No. of events/ participants	IR per 1000PY (95% CI)	10-year cumulative incidence (%, 95%CI)	Model 1, HR (95%CI)	P value	Model 2, HR (95%CI)	P value	Model 3, HR (95%CI)	P value
**All-cause dementia**	10,284/223,777	9.5 (9.4-9.7)	8.6 (8.4-8.8)						
A1 <30	7,307/180,073	8.4 (8.2-8.6)	8.1 (7.9-8.3)	1.00 (ref)		1.00 (ref)		1.00 (ref)	
A2 30-299	2,498/36,053	14.4 (13.9-15.0)	10.8 (10.3-11.2)	1.25 (1.20-1.31)	<0.001	1.25 (1.19-1.31)	<0.001	1.24 (1.18-1.30)	<0.001
A3 ≥300	479/7,651	13.5 (12.3-14.7)	8.2 (7.4-8.9)	1.37 (1.25-1.51)	<0.001	1.37 (1.25-1.50)	<0.001	1.38 (1.26-1.52)	<0.001
**Type-specific dementia**									
**Alzheimer's disease**	1,881/223,777	1.7 (1.7-1.8)	1.6 (1.5-1.7)						
A1 <30	1,511/180,073	1.7 (1.7-1.8)	1.7 (1.6-1.8)	1.00 (ref)		1.00 (ref)		1.00 (ref)	
A2 30-299	323/36,053	1.9 (1.7-2.1)	1.5 (1.3-1.7)	0.80 (0.71-0.90)	<0.001	0.85 (0.76-0.96)	<0.05	0.95 (0.84-1.07)	0.37
A3 ≥300	47/7,651	1.3 (1.0-1.8)	0.9 (0.6-1.1)	0.65 (0.49-0.88)	<0.001	0.73 (0.54-0.97)	<0.05	0.91 (0.68-1.22)	0.53
**Mixed dementia**	2,388/223,777	2.2 (2.1-2.3)	2.1 (2.0-2.2)						
A1 <30	1,728/180,073	2.0 (1.9-2.1)	2.0 (1.9-2.1)	1.00 (ref)		1.00 (ref)		1.00 (ref)	
A2 30-299	562/36,053	3.2 (3.0-3.5)	2.5 (2.3-2.8)	1.17 (1.06-1.28)	<0.001	1.16 (1.05-1.27)	<0.001	1.15 (1.04-1.27)	<0.05
A3 ≥300	98/7,651	2.8 (2.2-3.4)	1.8 (1.4-2.2)	1.17 (0.96-1.44)	0.12	1.15 (0.94-1.41)	0.17	1.20 (0.97-1.48)	0.09
**Vascular dementia**	2,359/223,777	2.2 (2.1-2.3)	2.1 (2.0-2.2)						
A1 <30	1,595/180,073	1.8 (1.7-1.9)	1.9 (1.7-2.0)	1.00 (ref)		1.00 (ref)		1.00 (ref)	
A2 30-299	647/36,053	3.7 (3.5-4.0)	3.0 (2.7-3.2)	1.49 (1.36-1.63)	<0.001	1.44 (1.31-1.58)	<0.001	1.33 (1.21-1.46)	<0.001
A3 ≥300	117/7,651	3.3 (2.7-3.9)	2.1 (1.7-2.5)	1.54 (1.28-1.86)	<0.001	1.46 (1.21-1.77)	<0.001	1.32 (1.09-1.60)	<0.05
**Lewy Body dementia and Parkinson's Disease with dementia**	261/223,777	0.2 (0.2-0.3)	0.2 (0.2-0.3)						
A1 <30	217/180,073	0.2 (0.2-0.3)	0.3 (0.2-0.3)	1.00 (ref)		1.00 (ref)		1.00 (ref)	
A2 30-299	40/36,053	0.2 (0.2-0.3)	0.2 (0.1-0.3)	0.77 (0.55-1.08)	0.13	0.71 (0.51-1.00)	0.05	0.78 (0.55-1.10)	0.16
A3 ≥300	4/7,651	0.1 (0.0-0.3)	0.1 (0.0-0.1)	0.42 (0.16-1.12)	0.08	0.37 (0.14-0.99)	0.05	0.41 (0.15-1.12)	0.08
**Frontotemporal dementia**	103/223,777	0.1 (0.1-0.1)	0.1 (0.1-0.1)						
A1 <30	80/180,073	0.1 (0.1-0.1)	0.1 (0.1-0.1)	1.00 (ref)		1.00 (ref)		1.00 (ref)	

A2 30-299	19/36,053	0.1 (0.1-0.2)	0.1 (0.0-0.1)	1.11 (0.67-1.83)	0.69	1.07 (0.65-1.78)	0.79	1.15 (0.69-1.92)	0.60
A3 ≥300	4/7,651	0.1 (0.0-0.3)	0.1 (0.0-0.1)	1.18 (0.43-3.23)	0.74	1.15 (0.42-3.15)	0.78	1.45 (0.51-4.08)	0.49
**Unspecified dementia**	3,010/223,777	2.8 (2.7-2.9)	2.6 (2.5-2.7)						
A1 <30	1,975/180,073	2.3 (2.2-2.4)	2.3 (2.2-2.4)	1.00 (ref)		1.00 (ref)		1.00 (ref)	
A2 30-299	844/36,053	4.9 (4.5-5.2)	3.8 (3.5-4.0)	1.52 (1.40-1.65)	<0.001	1.53 (1.40-1.66)	<0.001	1.49 (1.37-1.62)	<0.001
A3 ≥300	191/7,651	5.4 (4.6-6.2)	3.3 (2.8-3.8)	2.00 (1.73-2.32)	<0.001	2.02 (1.74-2.35)	<0.001	1.92 (1.64-2.24)	<0.001
**Other dementia**	282/223,777	0.3 (0.2-0.3)	0.2 (0.2-0.3)						
A1 <30	201/180,073	0.2 (0.2-0.3)	0.2 (0.2-0.3)	1.00 (ref)		1.00 (ref)		1.00 (ref)	
A2 30-299	63/36,053	0.4 (0.3-0.5)	0.3 (0.2-0.4)	1.28 (0.96-1.71)	0.09	1.19 (0.89-1.58)	0.24	1.23 (0.92-1.65)	0.17
A3 ≥300	18/7,651	0.5 (0.3-0.8)	0.3 (0.2-0.4)	1.99 (1.23-3.22)	<0.05	1.77 (1.09-2.88)	<0.05	1.80 (1.07-3.03)	<0.05

HRs and 95% confidence intervals were derived from Cox proportional hazards regression models.

The 10-year cumulative incidence was estimated using the Aalen-Johansen estimator, accounting for mortality as a competing event.

Model 1: adjusted for age;

model 2: model 1 + adjusted for sex, education, tobacco abuse, alcohol abuse, diagnosed obesity, cancer, depression, and hearing loss;

model 3: model 2 + adjusted for diabetes, hypertension, congestive heart failure, myocardial infarction, atrial fibrillation, stroke, ACEi/ARBs, beta blockers, calcium channel blockers, NSAIDs, statins, and eGFR.

Abbreviations: ACEi, angiotensin-converting enzyme inhibitor; ARB, angiotensin receptor blockers; eGFR, estimated glomerular filtration rate; HR, hazard ratio; IR, incidence rate; KDIGO, Kidney Disease Improving Global Outcomes; NSAIDs, nonsteroidal anti-inflammatory agents; PY, person-year.

Table S12. Associations of KDIGO albuminuria categories with the incidence of all-cause dementia and type-specific dementia, in the sensitivity analysis of starting the study follow-up from 2012.

KDIGO albuminuria categories, mg/g	No. of events/ participants	IR per 1000PY (95% CI)	Model 1, HR (95%CI)	P value	Model 2, HR (95%CI)	P value	Model 3, HR (95%CI)	P value
**All-cause dementia**	5,272/119,558	13.7 (13.3-14.0)						
A1 <30	3,642/93,076	12.1 (11.7-12.5)	1.00 (ref)		1.00 (ref)		1.00 (ref)	
A2 30-299	1,377/21,955	19.3 (18.3-20.4)	1.27 (1.19-1.35)	<0.001	1.27 (1.19-1.35)	<0.001	1.25 (1.17-1.33)	<0.001
A3 ≥300	253/4,527	17.7 (15.6-20.0)	1.22 (1.08-1.39)	<0.001	1.22 (1.08-1.39)	<0.001	1.25 (1.10-1.43)	<0.001
**Type-specific dementia**								
**Alzheimer's disease**	1,090/119,558	2.8 (2.7-3.0)						
A1 <30	860/93,076	2.9 (2.7-3.1)	1.00 (ref)		1.00 (ref)		1.00 (ref)	
A2 30-299	204/21,955	2.9 (2.5-3.3)	0.83 (0.71-0.97)	<0.05	0.89 (0.76-1.04)	0.14	0.99 (0.85-1.16)	0.95
A3 ≥300	26/4,527	1.8 (1.2-2.7)	0.55 (0.37-0.81)	<0.001	0.62 (0.42-0.92)	<0.05	0.81 (0.55-1.21)	0.31
**Mixed dementia**	1,249/119,558	3.2 (3.1-3.4)						
A1 <30	897/93,076	3.0 (2.8-3.2)	1.00 (ref)		1.00 (ref)		1.00 (ref)	
A2 30-299	300/21,955	4.2 (3.8-4.7)	1.12 (0.98-1.28)	0.10	1.11 (0.97-1.26)	0.14	1.07 (0.94-1.23)	0.30
A3 ≥300	52/4,527	3.6 (2.7-4.8)	1.02 (0.77-1.35)	0.89	1.00 (0.75-1.32)	0.99	0.99 (0.74-1.33)	0.97
**Vascular dementia**	1,211/119,558	3.1 (3.0-3.3)						
A1 <30	757/93,076	2.5 (2.3-2.7)	1.00 (ref)		1.00 (ref)		1.00 (ref)	
A2 30-299	379/21,955	5.3 (4.8-5.9)	1.68 (1.48-1.90)	<0.001	1.61 (1.42-1.82)	<0.001	1.47 (1.29-1.67)	<0.001
A3 ≥300	75/4,527	5.2 (4.1-6.6)	1.75 (1.38-2.22)	<0.001	1.63 (1.28-2.07)	<0.001	1.49 (1.17-1.91)	<0.001
**Lewy Body dementia and Parkinson's Disease with dementia**	141/119,558	0.4 (0.3-0.4)						
A1 <30	112/93,076	0.4 (0.3-0.4)	1.00 (ref)		1.00 (ref)		1.00 (ref)	
A2 30-299	24/21,955	0.3 (0.2-0.5)	0.83 (0.53-1.29)	0.41	0.78 (0.50-1.22)	0.27	0.87 (0.55-1.38)	0.56
A3 ≥300	5/4,527	0.3 (0.1-0.8)	0.88 (0.36-2.17)	0.79	0.79 (0.32-1.95)	0.62	0.99 (0.39-2.52)	0.98
**Frontotemporal dementia**	53/119,558	0.1 (0.1-0.2)						
A1 <30	38/93,076	0.1 (0.1-0.2)	1.00 (ref)		1.00 (ref)		1.00 (ref)	
A2 30-299	10/21,955	0.1 (0.1-0.3)	1.08 (0.54-2.18)	0.83	1.06 (0.52-2.14)	0.88	1.14 (0.56-2.34)	0.72

A3 ≥300	5/4,527	0.3 (0.1-0.8)	2.72 (1.07-6.93)	<0.05	2.65 (1.03-6.81)	<0.05	3.21 (1.16-8.84)	<0.05
**Unspecified dementia**	1,386/119,558	3.6 (3.4-3.8)						
A1 <30	879/93,076	2.9 (2.7-3.1)	1.00 (ref)		1.00 (ref)		1.00 (ref)	
A2 30-299	426/21,955	6.0 (5.4-6.6)	1.54 (1.37-1.73)	<0.001	1.55 (1.38-1.75)	<0.001	1.50 (1.33-1.69)	<0.001
A3 ≥300	81/4,527	5.7 (4.5-7.0)	1.56 (1.24-1.96)	<0.001	1.58 (1.26-1.99)	<0.001	1.53 (1.21-1.94)	<0.001
**Other dementia**	142/119,558	0.4 (0.3-0.4)						
A1 <30	99/93,076	0.3 (0.3-0.4)	1.00 (ref)		1.00 (ref)		1.00 (ref)	
A2 30-299	34/21,955	0.5 (0.3-0.7)	1.20 (0.81-1.77)	0.37	1.10 (0.74-1.64)	0.63	1.12 (0.75-1.68)	0.57
A3 ≥300	9/4,527	0.6 (0.3-1.2)	1.65 (0.83-3.27)	0.15	1.43 (0.72-2.85)	0.3	1.44 (0.70-2.97)	0.33

HRs and 95% confidence intervals were derived from Cox proportional hazards regression models.

Model 1: adjusted for age;

model 2: model 1 + adjusted for sex, education, tobacco abuse, alcohol abuse, diagnosed obesity, cancer, depression, and hearing loss;

model 3: model 2 + adjusted for diabetes, hypertension, congestive heart failure, myocardial infarction, atrial fibrillation, stroke, ACEi/ARBs, beta blockers, calcium channel blockers, NSAIDs, statins, and eGFR.

Abbreviations: ACEi, angiotensin-converting enzyme inhibitor; ARB, angiotensin receptor blockers; eGFR, estimated glomerular filtration rate; HR, hazard ratio; IR, incidence rate; KDIGO, Kidney Disease Improving Global Outcomes; NSAIDs, nonsteroidal anti-inflammatory agents; PY, person-year.

Table S13. Associations of KDIGO albuminuria categories with the incidence of dementia in the sensitivity analysis of handling mortality as a competing event.

KDIGO albuminuria categories, mg/g	No. of events/participants	HR for dementia (95%CI)	sHR for dementia (95%CI)	HR for mortality (95%CI)
**All-cause dementia**	9,435/132,869			
A1 <30	6,663/102,831	1.00 (ref)	1.00 (ref)	1.00 (ref)
A2 30-299	2,341/24,908	1.25 (1.19-1.31)	1.09 (1.04-1.15)	1.71 (1.66-1.76)
A3 ≥300	431/5,130	1.37 (1.23-1.51)	1.00 (0.90-1.11)	2.54 (2.43-2.66)
A2 & A3	2,772/30,038	1.26 (1.21-1.32)	1.08 (1.03-1.13)	1.83 (1.79-1.88)

HRs and 95% confidence intervals were derived from Cox proportional hazards regression models.

sHRs and 95% confidence intervals were derived from Fine and Gray hazards regression models.

Models are adjusted for age, sex, education, tobacco abuse, alcohol abuse, diagnosed obesity, diabetes, hypertension, congestive heart failure, myocardial infarction, atrial fibrillation, stroke, cancer, depression, hearing loss, ACEi/ARBs, beta blockers, calcium channel blockers, NSAIDs, statins, and eGFR.

Abbreviations: ACEi, angiotensin-converting enzyme inhibitor; ARB, angiotensin receptor blockers; eGFR, estimated glomerular filtration rate; HR, hazard ratio; KDIGO, Kidney Disease Improving Global Outcomes; NSAIDs, nonsteroidal anti-inflammatory agents; sHR, sub-distribution hazard ratio.

Figure S1. Flow chart of study participants and study design.

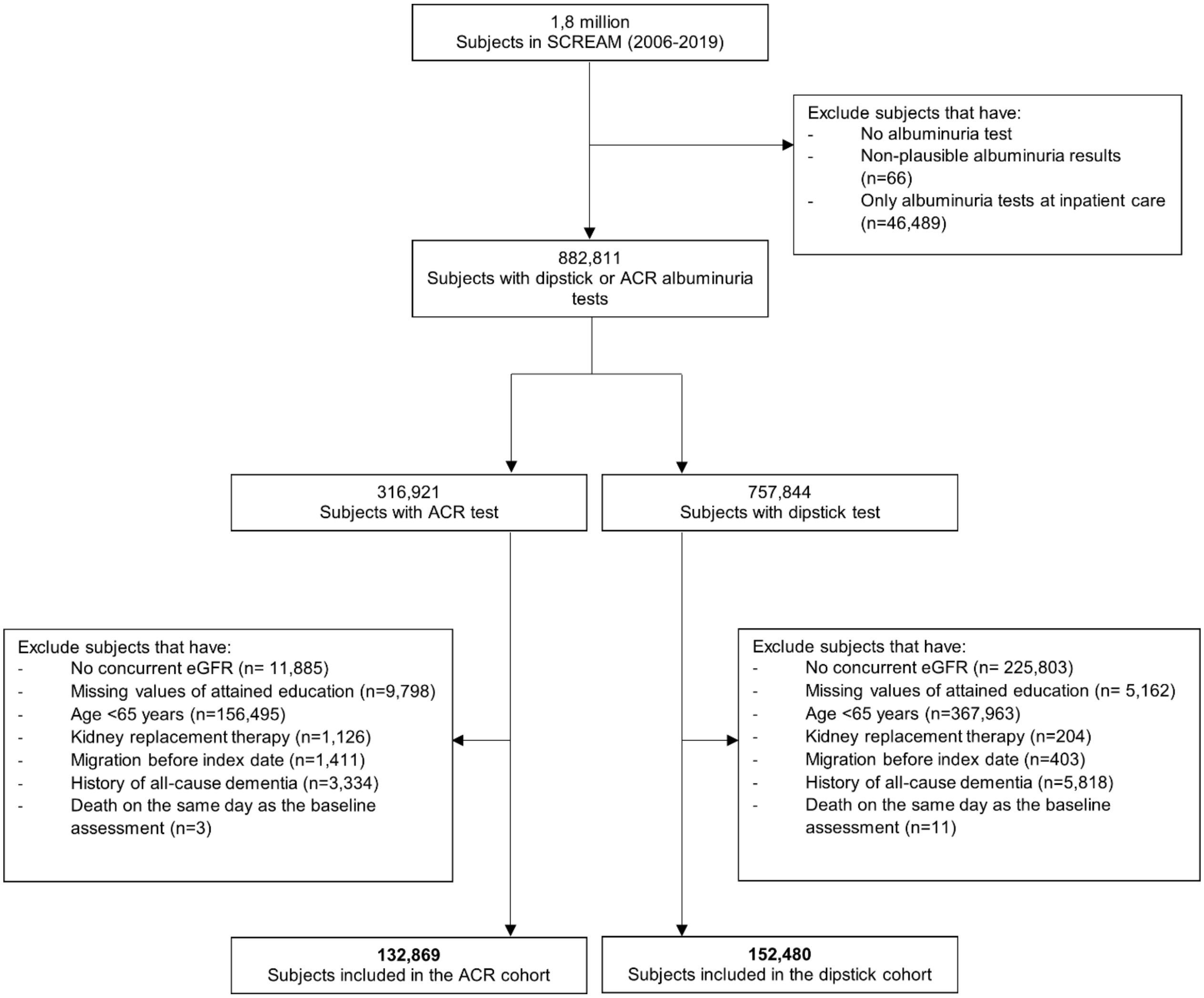

Non-plausible albuminuria results refer to measurements that report negative values.

Abbreviations: ACR, urine albumin-creatinine ratio; eGFR, estimated glomerular filtration rate; SCREAM, the Stockholm Creatinine project.

Figure S2. Assessment of the proportional hazards assumption by the Schoenfeld residuals for the Cox model examining the risk of all-cause dementia.

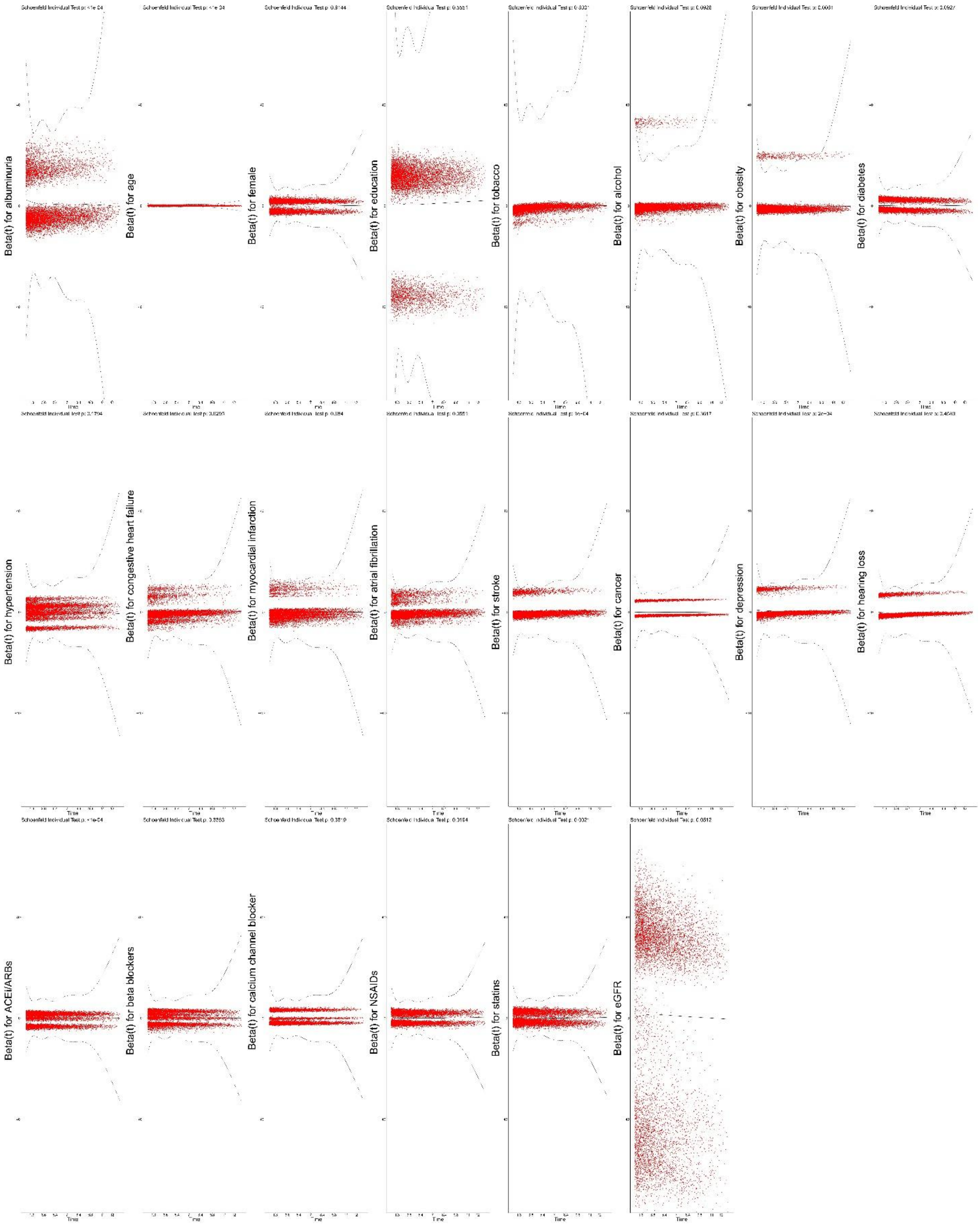

The graphs show Scaled Schoenfeld residuals plotted against transformed time for each covariate in the Cox proportional hazards model. The Cox proportional hazards regression model examined the association between KDIGO albuminuria categories and all-cause dementia, adjusted for age, sex, education, tobacco abuse, alcohol abuse, diagnosed obesity, diabetes, hypertension, congestive heart failure, myocardial infarction, atrial fibrillation, stroke, cancer, depression, hearing loss, ACEi/ARBs, beta blockers, calcium channel blockers, NSAIDs, statins, and eGFR. Abbreviations: ACEi, angiotensin-converting enzyme inhibitor; ARB, angiotensin receptor blockers; eGFR, estimated glomerular filtration rate; KDIGO, Kidney Disease Improving Global Outcomes; NSAIDs, nonsteroidal anti-inflammatory agents.

Figure S3. Adjusted hazard ratio of albuminuria with the incidence of all-cause dementia (panel A) and distribution of albuminuria (panel B), with the reference standard for albuminuria being 30mg/g.

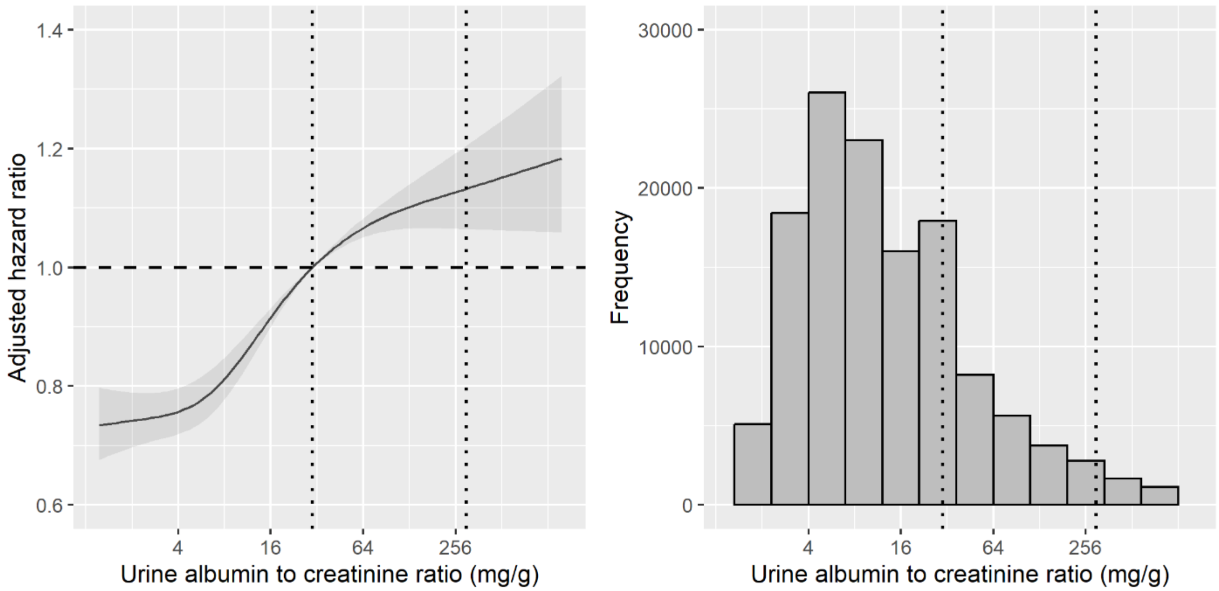

The spline shows the association of albuminuria with the incidence of all-cause dementia. Data were fitted by Cox proportional hazards regression models based upon restricted cubic splines with 4 knots, and adjusted for age, sex, education, tobacco abuse, alcohol abuse, diagnosed obesity, diabetes, hypertension, congestive heart failure, myocardial infarction, atrial fibrillation, stroke, cancer, depression, hearing loss, ACEi/ARBs, beta blockers, calcium channel blockers, NSAIDs, statins, and eGFR. The spline curves are truncated at the 1.0th and 99.0th percentile of the distribution curve. The reference standard for albuminuria is 30 mg/g. The dashed lines represent 30mg/g and 300 mg/g. P value for the nonlinear association is P <0.001. Abbreviations: ACEi, angiotensin-converting enzyme inhibitor; ARB, angiotensin receptor blockers; eGFR, estimated glomerular filtration rate; NSAIDs, nonsteroidal anti-inflammatory agents.

Figure S4. Adjusted hazard ratio of albuminuria with the incidence of type-specific dementia.

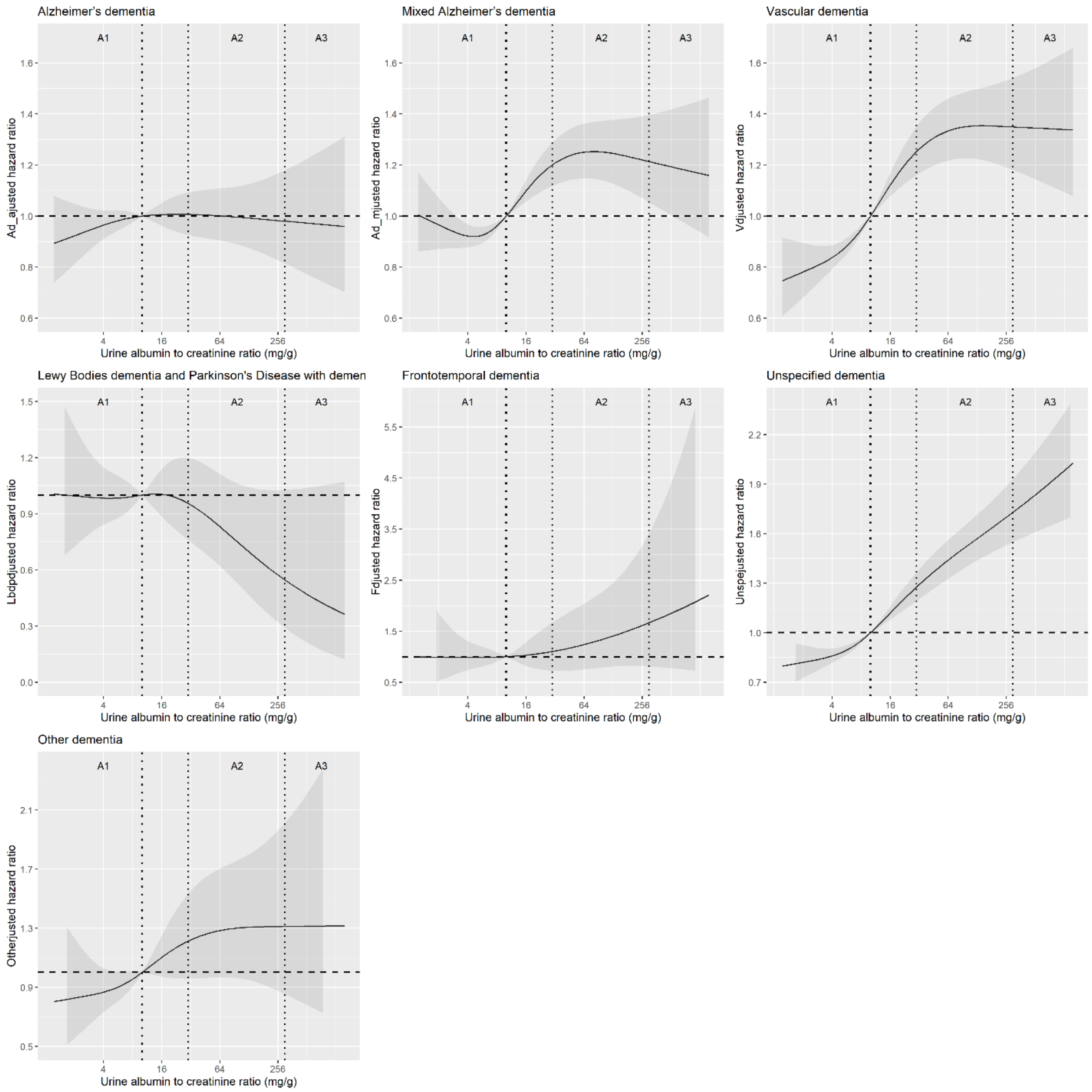

The splines show the association of albuminuria with the incidence of type-specific dementia. Data were fitted by Cox proportional hazards regression models based upon restricted cubic splines with 4 knots, and adjusted for age, sex, education, tobacco abuse, alcohol abuse, diagnosed obesity, diabetes, hypertension, congestive heart failure, myocardial infarction, atrial fibrillation, stroke, cancer, depression, hearing loss, ACEi/ARBs, beta blockers, calcium channel blockers, NSAIDs, statins, and eGFR. The spline curves are truncated at the 1.0th and 99.0th percentile of the distribution curve. The reference standard for albuminuria is 10 mg/g. The

dashed lines represent 30mg/g and 300 mg/g. Except for frontotemporal dementia ($P_{non\text{-}linearity}$ =0.68), P values for the nonlinear associations of albuminuria with the incidence of all type-specific dementia are P <0.001. Abbreviations: ACEi, angiotensin-converting enzyme inhibitor; ARB, angiotensin receptor blockers; eGFR, estimated glomerular filtration rate; NSAIDs, nonsteroidal anti-inflammatory agents.

Figure S5. Adjusted hazard ratio of albuminuria with the incidence of type-specific dementia, with the reference standard for albuminuria being 30mg/g.

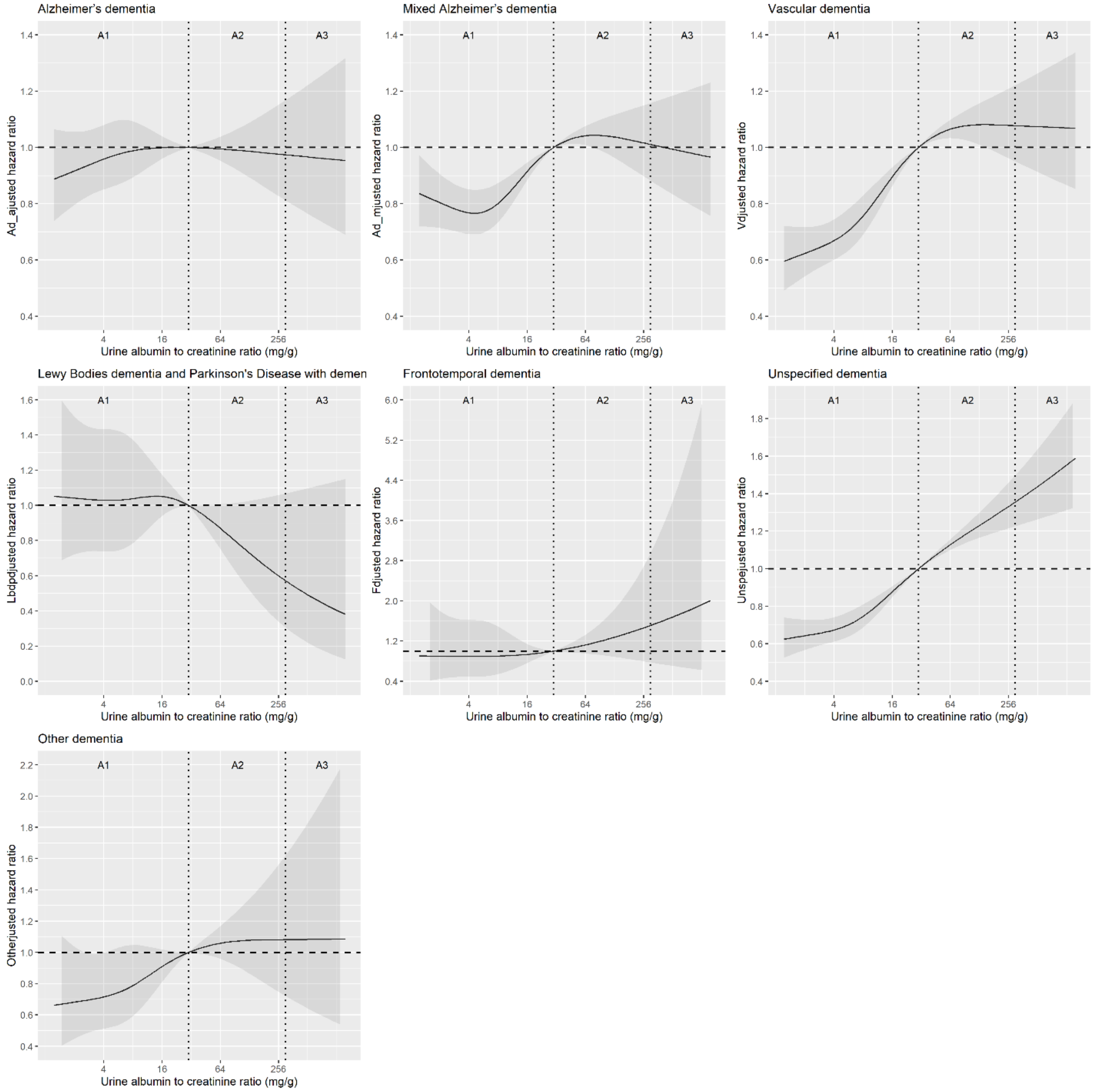

The splines show the association of albuminuria with the incidence of type-specific dementia. Data were fitted by Cox proportional hazards regression models based upon restricted cubic splines with 4 knots, and adjusted for age, sex, education, tobacco abuse, alcohol abuse, diagnosed obesity, diabetes, hypertension, congestive heart failure, myocardial infarction, atrial fibrillation, stroke, cancer, depression, hearing loss, ACEi/ARBs, beta blockers, calcium channel blockers, NSAIDs, statins, and eGFR. The spline curves are truncated at the 1.0th and 99.0th

percentile of the distribution curve. The reference standard for albuminuria is 30 mg/g. The dashed lines represent 30mg/g and 300 mg/g. Except for frontotemporal dementia ($P_{non\text{-}linearity}$ =0.68), P values for the nonlinear associations of albuminuria with the incidence of all type-specific dementia are P <0.001. Abbreviations: ACEi, angiotensin-converting enzyme inhibitor; ARB, angiotensin receptor blockers; eGFR, estimated glomerular filtration rate; NSAIDs, nonsteroidal anti-inflammatory agents.

Figure S6. Subgroup analyses investigating effect modification of the association of KDIGO albuminuria categories (30-299, and ≥300 versus <30 mg/g) with the incidence of dementia by age, sex, hypertension, diabetes, and eGFR.

	n/N	95% CI	P for interaction
**Age, <75 years**			
ACR <30	2,232/62,009	1.00 (ref)	
ACR 30-299	587/11,139	1.26 [1.15; 1.38]	0.44
ACR ≥300	116/2,300	1.25 [1.03; 1.52]	0.69
**Age, <75 years**			
ACR <30	4,431/40,822	1.00 (ref)	
ACR 30-299	1,754/13,769	1.25 [1.18; 1.32]	
ACR ≥300	315/2,830	1.38 [1.23; 1.56]	
**Male**			
ACR <30	2,734/48,396	1.00 (ref)	
ACR 30-299	1,152/14,086	1.19 [1.11; 1.28]	0.11
ACR ≥300	263/3,298	1.34 [1.17; 1.53]	0.90
**Female**			
ACR <30	3,929/54,435	1.00 (ref)	
ACR 30-299	1,189/10,822	1.30 [1.22; 1.39]	
ACR ≥300	168/1,832	1.39 [1.19; 1.63]	
**Hypertension, no**			
ACR <30	1,199/17,777	1.00 (ref)	
ACR 30-299	276/2,692	1.21 [1.06; 1.39]	0.67
ACR ≥300	33/393	1.19 [0.83; 1.70]	0.50
**Hypertension, yes**			
ACR <30	5,464/85,054	1.00 (ref)	
ACR 30-299	2,065/22,216	1.25 [1.19; 1.32]	
ACR ≥300	398/4,737	1.39 [1.25; 1.54]	
**Diabetes, no**			
ACR <30	3,856/71,483	1.00 (ref)	
ACR 30-299	1,198/14,733	1.28 [1.20; 1.37]	0.09
ACR ≥300	210/2,795	1.41 [1.22; 1.63]	0.40
**Diabetes, yes**			
ACR <30	2,807/31,348	1.00 (ref)	
ACR 30-299	1,143/10,175	1.21 [1.12; 1.30]	
ACR ≥300	221/2,335	1.32 [1.14; 1.52]	
**eGFR, <60 ml/min/1.73m²**			
ACR <30	1,741/20,719	1.00 (ref)	
ACR 30-299	942/9,510	1.24 [1.14; 1.34]	0.38
ACR ≥300	282/3,330	1.38 [1.21; 1.57]	0.48
**eGFR, ≥60 ml/min/1.73m²**			
ACR <30	4,922/82,112	1.00 (ref)	
ACR 30-299	1,399/15,398	1.24 [1.17; 1.32]	
ACR ≥300	149/1,800	1.28 [1.08; 1.50]	

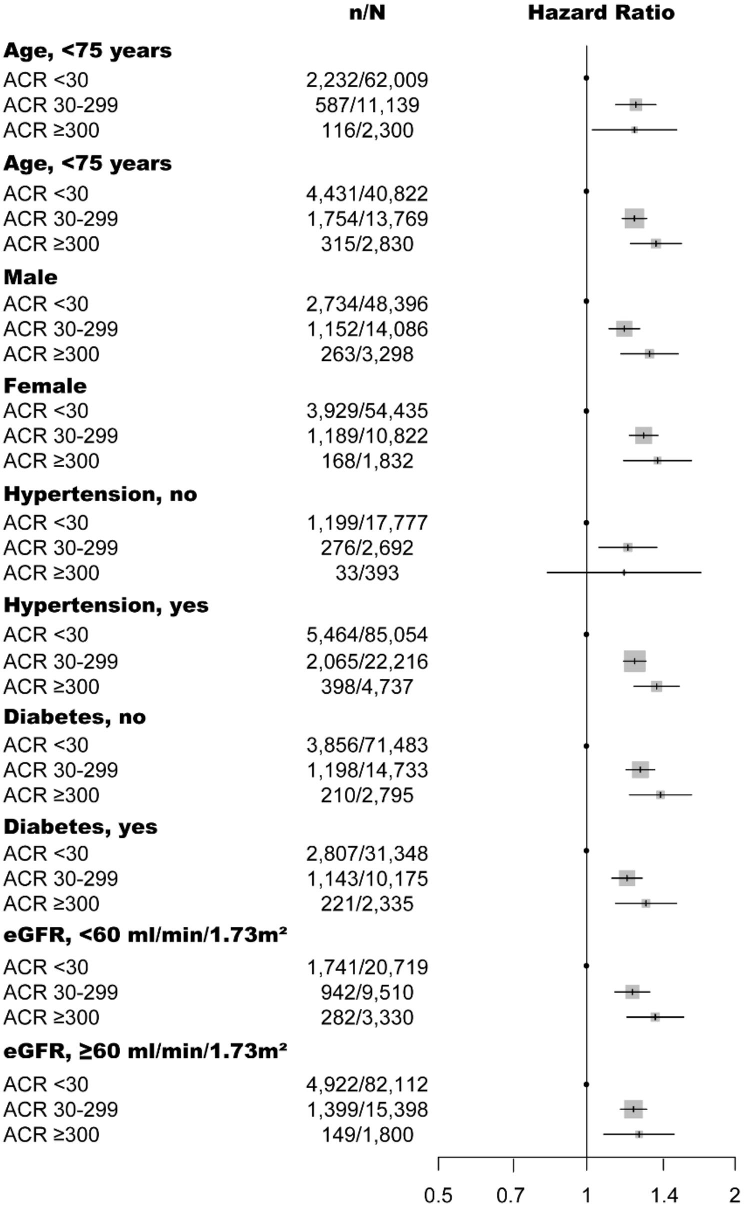

HRs and 95% CIs were derived from Cox proportional hazards regression models.

HRs were adjusted for age, sex, education, tobacco abuse, alcohol abuse, diagnosed obesity, diabetes, hypertension, congestive heart failure, myocardial infarction, atrial fibrillation, stroke, cancer, depression, hearing loss, ACEi/ARBs, beta blockers, calcium channel blockers, NSAIDs, statins, and eGFR.

Abbreviations: ACEi, angiotensin-converting enzyme inhibitor; ARB, angiotensin receptor blockers; eGFR, estimated glomerular filtration rate; HR, hazard ratio; KDIGO, Kidney Disease Improving Global Outcomes; NSAIDs, nonsteroidal anti-inflammatory agents.
